# Piezoelectric stimulation enhances bone regeneration in alveolar bone defects through metabolic reprogramming of macrophages

**DOI:** 10.1002/EXP.20230149

**Published:** 2024-06-10

**Authors:** Baiyan Sui, Tingting Ding, Xingyi Wan, Yuxiao Chen, Xiaodi Zhang, Yuanbo Cui, Jie Pan, Linlin Li, Xin Liu

**Affiliations:** ^1^ Department of Dental Materials Shanghai Biomaterials Research and Testing Center Shanghai Ninth People's Hospital, Shanghai Jiao Tong University School of Medicine; College of Stomatology, Shanghai Jiao Tong University; National Center for Stomatology; National Clinical Research Center for Oral Diseases; Shanghai Key Laboratory of Stomatology Shanghai China; ^2^ Beijing Institute of Nanoenergy and Nanosystems Chinese Academy for Sciences Beijing China; ^3^ School of Nanoscience and Engineering University of Chinese Academy of Sciences Beijing China; ^4^ Institute for Cell Engineering Department of Neurology Johns Hopkins University School of Medicine Baltimore Maryland USA; ^5^ Leavey School of Business Santa Clara University Santa Clara California USA; ^6^ Department of Orthodontics, Shanghai Stomatological Hospital and School of Stomatology Fudan University Shanghai China

**Keywords:** bone regeneration, macrophage reprogramming, piezoelectric stimulation

## Abstract

Immunomodulation has emerged as a promising strategy for promoting bone regeneration. However, designing osteoimmunomodulatory biomaterial that can respond to mechanical stress in the unique microenvironment of alveolar bone under continuous occlusal stress remains a significant challenge. Herein, a wireless piezoelectric stimulation system, namely, piezoelectric hydrogel incorporating BaTiO_3_ nanoparticles (BTO NPs), is successfully developed to generate piezoelectric potentials for modulating macrophage reprogramming. The piezoelectric stimulation reprograms macrophages towards the M2 phenotype, which subsequently induces osteogenic differentiation of bone marrow mesenchymal stem cells (BMSCs). RNA sequencing analysis reveals that piezoelectricity‐modulated macrophage M2 polarization is closely associated with metabolic reprogramming, including increased amino acid biosynthesis and fatty acid oxidation. The composite hydrogel with excellent biocompatibility exhibits immunomodulatory and osteoinductive activities. In a rat model of alveolar bone defects, the piezoelectric hydrogel effectively promotes endogenous bone regeneration at the load‐bearing sites. The piezoelectric‐driven osteoimmunomodulation proposed in this study not only broadens understanding of the mechanism underlying piezoelectric biomaterials for tissue regeneration but also provides new insights into the design and development of next‐generation immunomodulatory biomaterials.

## Graphical abstract

BTO NPs‐mediated piezoelectric stimulation effectively induced macrophage M2 polarization and metabolic reprogramming, represented by enhanced TCA cycle and one‐carbon folate cycle. After that, anti‐inflammatory and pro‐differentiation M2 promoted osteogenic differentiation of bone marrow mesenchymal stem cells. The osteo‐friendly immune microenvironment formed by piezoelectric stimulation effectively promoted the regeneration of alveolar bone defects.

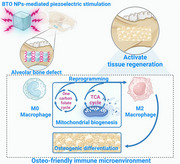

## 1 INTRODUCTION

Regeneration of alveolar bone defects caused by trauma, tumor, or infection presents a significant clinical challenge due to the continuous resorption of the alveolar ridge, limited residual bone volume, and restricted self‐healing potential.^[^ ^1^ ^]^ Recent studies have demonstrated the role of immunomodulation, particularly macrophage reprogramming, in accelerating bone regeneration by creating a favorable osteoimmune microenvironment.^[^ ^2^ ^]^ Given this, various strategies have been developed to explore osteo‐immunomodulatory biomaterials, such as cytokines/bioactive ion‐doped materials and surface modifications (e.g., topography/architecture, wettability, or charge), to shift macrophages from a pro‐inflammatory M1 phenotype to a pro‐regenerative/anti‐inflammatory M2 phenotype.^[^ ^2^ ^]^ However, the success of macrophage reprogramming heavily relies on tissue‐specific microenvironments and niches.^[^ ^3^ ^]^ Current research primarily focuses on the effects of physicochemical properties of biomaterials or exogenous bioactive factors, often neglecting the potential impact of mechanical or electrophysiological microenvironments, despite accumulating evidence suggesting the unique mechanical and immune microenvironment of alveolar bone induced by continuous occlusal stress stimuli.^[^ ^4^ ^]^

Recently, the importance of bioelectric signals in various biological processes such as embryogenesis, wound healing, and tissue regeneration has become increasingly evident.^[^ ^5^ ^]^ These signals play a crucial role in cell cycle regulation, migration, proliferation, and differentiation. Moreover, functional electric stimulation has been utilized for the modulation of neural communication, cardiomyocyte contraction, and osteogenic differentiation.^[^ ^5^, ^6^ ^]^ However, the inconvenience of using external power supplies limits their practical applications.^[^ ^7^ ^]^ Piezoelectric biomaterials have emerged as a promising alternative as they can generate piezoelectric potential in response to mechanical stress. This inherent property of piezoelectric biomaterials enables them to wirelessly promote cell migration, differentiation, and proliferation.^[^ ^5^, ^8^ ^]^ They have shown potential applications in bone healing acceleration through the modulation of macrophage phenotype switching.^[^ ^9^ ^]^ Despite these advancements, there are controversial findings regarding the effects of piezoelectric stimulation on macrophage polarization. A recent study reported that a piezoelectric film combined with ultrasonic irradiation induced M1 polarization in macrophages, leading to an enhanced proinflammatory response.^[^ ^10^ ^]^ Up to now, there still needs to be a more comprehensive understanding regarding the metabolic response of macrophages to piezoelectric stimulation.

**SCHEME 1:**
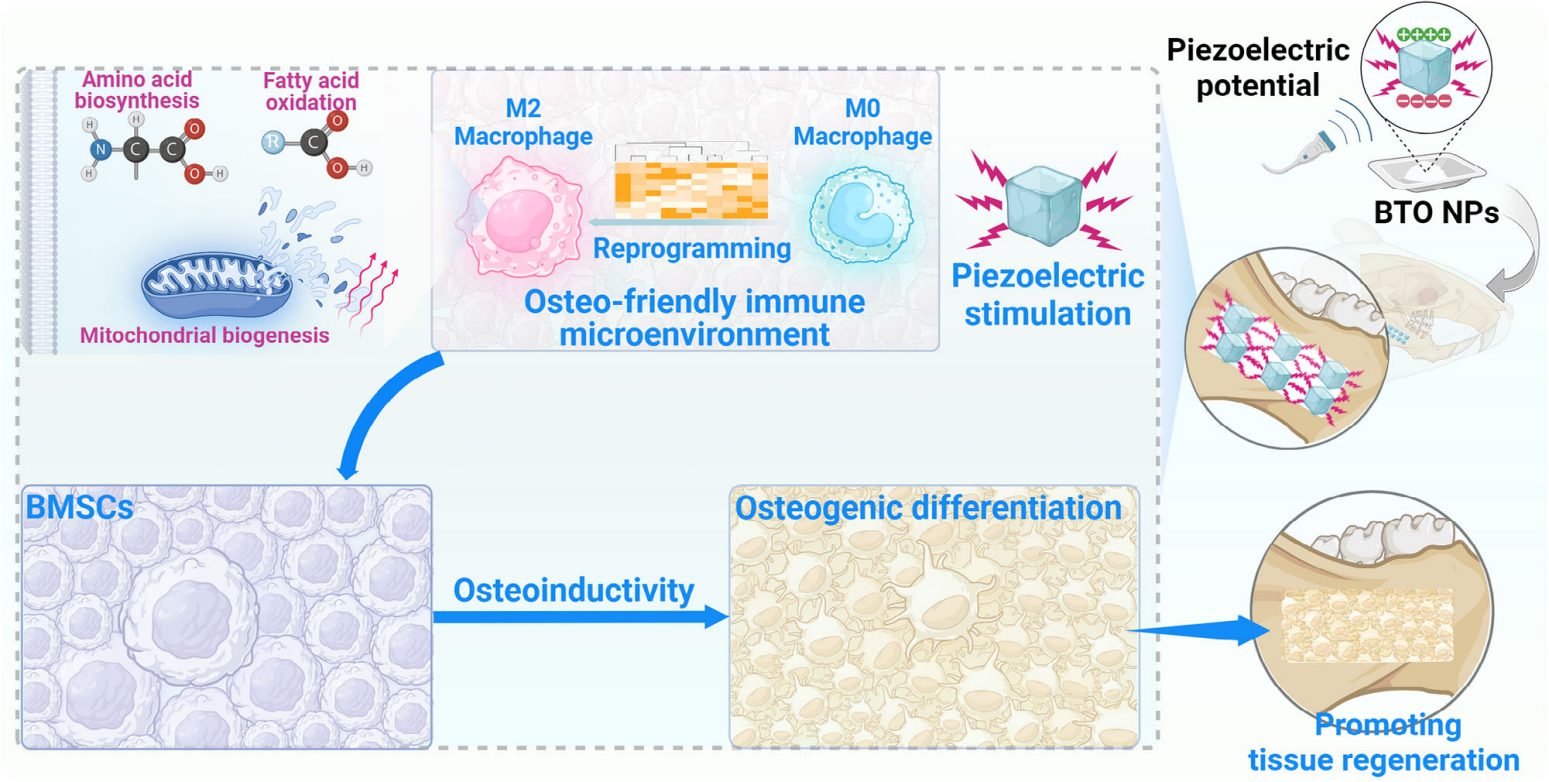
Piezoelectric stimulation promotes osteogenic differentiation of BMSCs and alveolar bone tissue regeneration via macrophage reprogramming.

Herein, we investigated the modulatory effects of piezoelectric stimulation on macrophages and its subsequent impact on bone marrow mesenchymal stem cells (BMSCs). To achieve this, we fabricated piezoelectric BTO NPs capable of generating surface piezoelectric potentials when subjected to mechanical stress. Furthermore, we employed gelatin methacryloyl (GelMA), a biocompatibility material with rapid shape adaptability, to fabricate a composite hydrogel with piezoelectric properties for repairing alveolar bone defects in rats (Scheme 1). In response to ultrasonic simulated stress, our piezoelectric BTO NPs were able to polarize M0 macrophages towards a pro‐regenerative M2 phenotype and subsequently promote the osteogenic differentiation of BMSCs via their immunomodulatory activities. To gain insights into the underlying mechanisms, we performed RNA sequencing analysis, which revealed that piezoelectric stimulation drove macrophage M2 polarization and metabolic reprogramming, enhancing amino acid biosynthesis and fatty acid oxidation, thereby facilitating mitochondrial biogenesis. Importantly, the BTO‐embedded composite hydrogels effectively promoted tissue regeneration in load‐bearing bone defects. These findings highlight the potential of piezoelectric stimulation as a promising strategy to enhance tissue regeneration through macrophage reprogramming.

## 2 RESULTS AND DISCUSSION

### 2.1 Fabrication and characterization of piezoelectric BTO NPs

**FIGURE 1:**
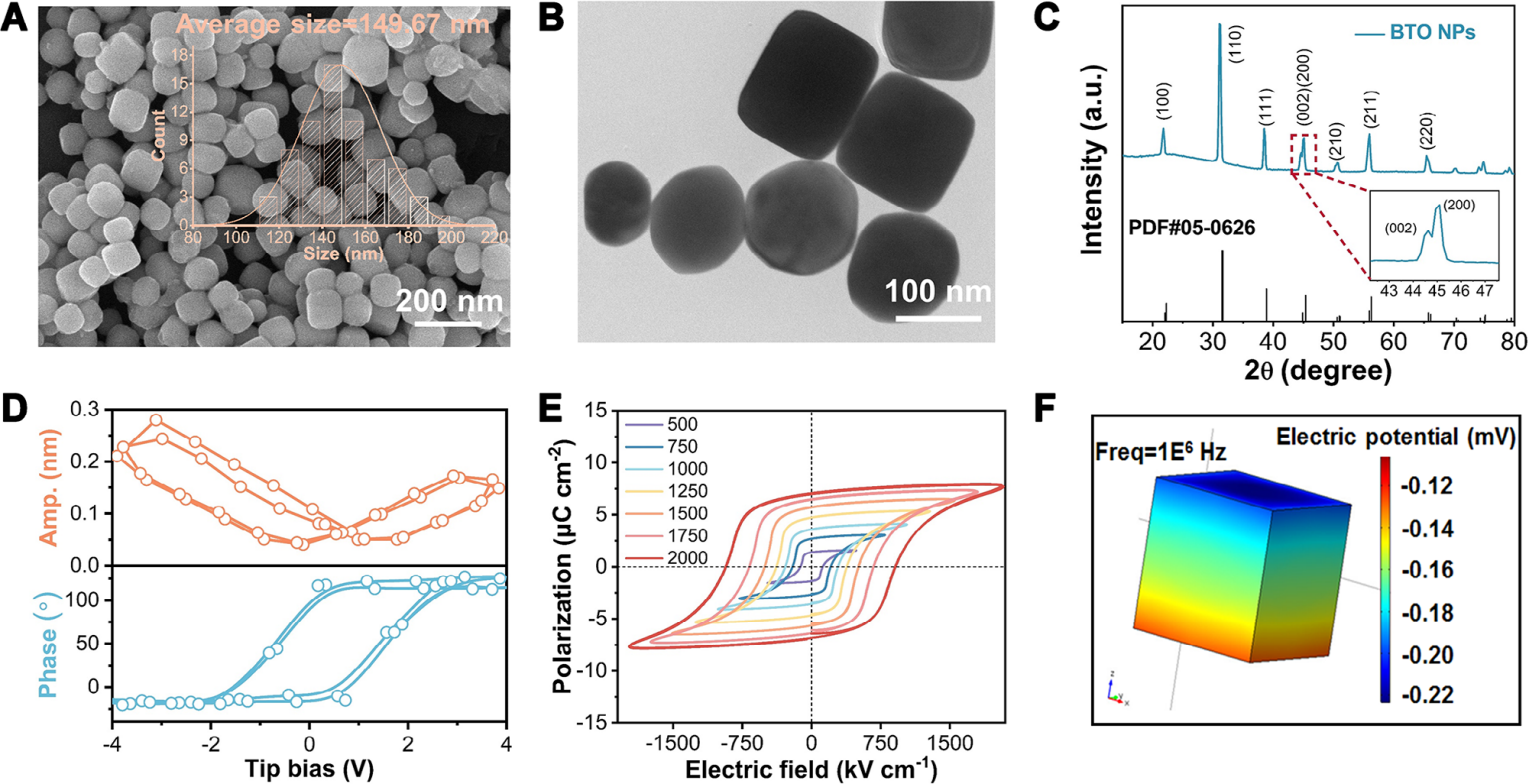
Characterization of BaTiO_3_ nanoparticles (BTO NPs). (A) SEM image and statistical size distribution (inset). (B) TEM image. (C) XRD pattern and corresponding standard JCPDS data. Inset shows the enlarged splitting peaks of (002, 200). (D) Piezoresponsive amplitude and phase curve obtained during two voltage switching cycles. (E) P–E hysteresis loops under different electric fields. (F) COSMOL stimulation of BTO NP generated piezoelectric potential under ultrasound stimulation.

In order to generate electrical signals when subjected to mechanical stimulation, we fabricated piezoelectric tetragonal BTO NPs. The fabricated BTO NPs exhibited a regular cubic shape with round corners, measuring approximately 149.67 ± 52.18 nm in size (Figure 1A,B). X‐ray diffraction (XRD) analysis confirmed that the NPs consisted solely of the tetragonal phase (Figure 1C, PDF#05‐0626).^[^ ^11^ ^]^ The piezoresponse force microscopy (PFM) results displayed standard butterfly‐shaped amplitude and phase curves (Figure 1D), indicating piezoelectricity of BTO NPs. The amplitude curve exhibited distinct variations in response to an applied external field, attributed to the changing strain. Additionally, the phase curve formed a hysteresis loop with 180° switching, proving the piezoelectric property of BTO NPs. The results of P–E measurements to investigate the ferroelectric phase suggested typical ferroelectric hysteresis behavior (Figure 1E). The remnant polarization (*P*_r_) and saturated polarization (*P*_s_) values increased until they reached maximum values of 7.07 µC cm^−2^ and 8 µC cm^−2^, respectively. The shape of the P–E loop gradually saturated with an increasing electric field, confirming the ferroelectric nature of BTO NPs. Additional characterizations, such as the HRTEM image and the corresponding SAED pattern, EDX mapping, and Raman scanning, further confirmed the tetragonal phase, elemental composition, and piezoelectric properties of the BTO NPs (Figures S1–S3, Supporting Information).

We explored COMSOL Multiphysics software to simulate the generated piezoelectric potential on the BTO NPs under mechanical stress. With an ultrasound intensity of 1 W cm^−2^ and a frequency of 1 MHz, an ultrasonic force of 10^6^ Pa was applied on the BTO NP surface. As a result, BTO NP generated a piezoelectric potential of approximately −0.12 mV and −0.22 mV on the two opposite surfaces, resulting in a net potential of approximately 0.1 mV (Figure 1F).

### 2.2 Macrophage reprogramming by piezoelectric stimulation

Macrophages play a crucial role in maintaining the delicate balance between inflammation and tissue regeneration through their ability to switch between different phenotypes.^[^ ^12^ ^]^ Depending on specific stimuli, macrophages can polarize into pro‐inflammatory M1 phenotype or anti‐inflammatory M2 phenotype.^[^ ^13^ ^]^ In this study, we investigated whether the piezoelectric stimulation generated by BTO NPs under mechanical stress can effectively modulate the macrophage phenotypes, thereby creating a favorable immune microenvironment to promote bone regeneration.

**FIGURE 2:**
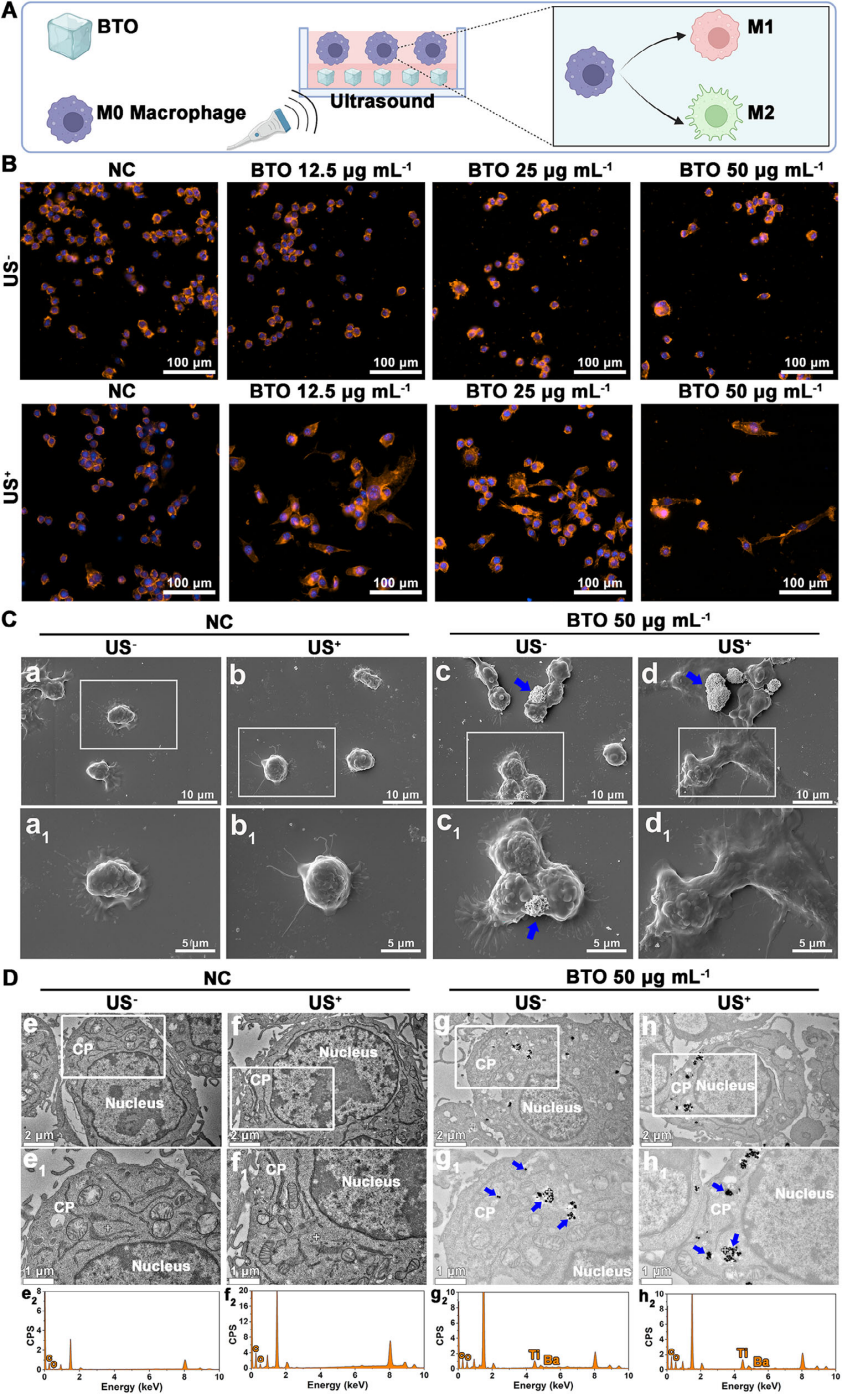
Macrophage morphological variability mediated by BTO NPs in vitro. (A) Schematic diagram of BTO NPs‐mediated RAW 264.7 cells polarization in vitro. (B) Representative fluorescence image of RAW 264.7 cells exposed to different concentrations BTO with/without ultrasound irritation (US^+^/US^−^) at 3 days. Phalloidin‐iFluorTM represents F‐actin (Tangerine), and DAPI represents cell nucleus (Blue). (C) SEM micrographs of RAW 264.7 cells morphology: RAW 264.7 cells were exposed to NC or 50 µg mL^−1^ BTO NPs with/without ultrasound irritation (US^+^/US^−^). (a_1_–d_1_) Higher magnification of the white pane regions in (a–d). (D) TEM images for intracellular localization of NPs: RAW 264.7 cells were exposed to NC or 50 µg mL^−1^ BTO NPs with/without ultrasound irritation (US^+^/US^−^). (e_1_–h_1_) Higher magnification of the white pane regions in (e–h), (e_2_–h_2_) graph of EDX analysis of the crosses in (e_1_–h_1_). CP: Cytoplasm, the blue arrow denoted BTO NPs. The RAW 264.7 cells cultured with cell medium were set as NC.

We assessed the cytotoxicity of BTO NPs on RAW 264.7 cells and determined the safe dosage range of 50–1000 µg mL^−1^ (Figure S4, Supporting Information). Subsequently, we evaluated the impact of the piezoelectric effect on macrophage phenotypes by examining changes in cell morphology,^[^ ^14^ ^]^ cell surface markers (CD 86 for M1 macrophages and CD 163 for M2 macrophages), and expression levels of typical marker genes (iNOs for M1 macrophages; Arginase‐1, YM1, and Fizz1 for M2 macrophages) after incubation of RAW 264.7 cells exposed to BTO NPs with or without ultrasound irritation (Figure 2A).

As shown in Figure 2B, Phalloidin was used to stain RAW 264.7 cells for F‐actin as representative of the cytoskeleton. The results demonstrated that RAW 264.7 cells stimulated with BTO NPs alone maintained their spherical morphology. In contrast, when treated with BTO NPs combined with the US, the cells exhibited a progressively elongated morphology as the concentration of BTO NPs increased (Figure 2B). Additionally, SEM analysis revealed significant directional spreading and flattening of cells under 50 µg mL^−1^ BTO NPs with US treatment, indicating polarization towards the M2 phenotype. In contrast, cells in the other control groups maintained a round morphology without polarization (Figure 2C). Further, TEM images showed the presence of agglomerated nano‐sized particles in the cytoplasm of RAW 264.7 cells exposed to BTO NPs, and EDX analysis confirmed that the agglomerated particles appeared as Ti and Ba peaks, which was not observed in the cells without BTO NPs treatment (Figure 2D). These findings suggested that BTO NPs can be endocytosed and localized in the cytoplasm of RAW 264.7 cells.

**FIGURE 3:**
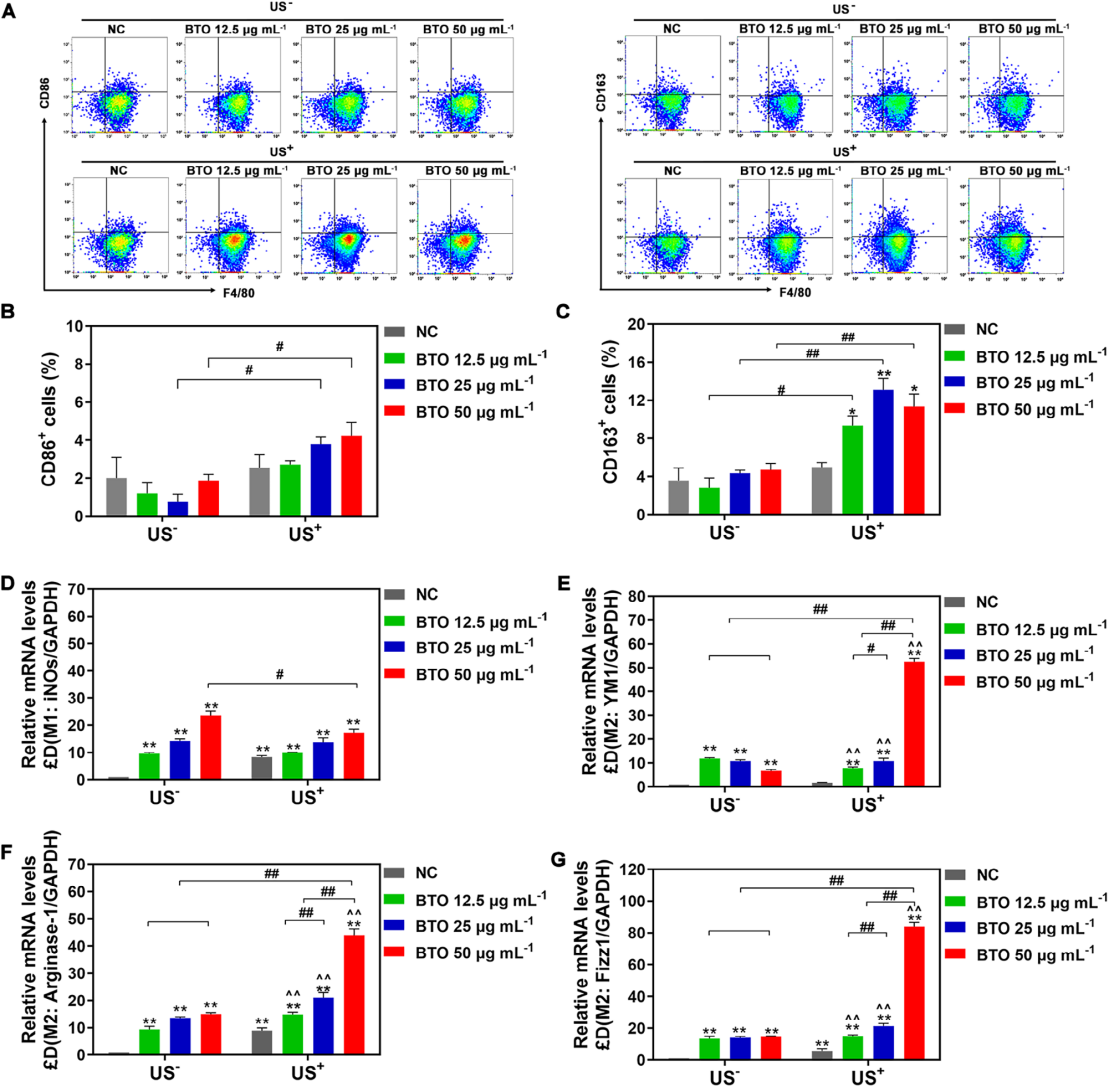
Macrophage polarization mediated by BTO NPs in vitro. (A–C) Flow cytometry analysis of RAW 264.7 cells polarization exposed to different concentrations of BTO NPs with/without ultrasound irritation (US^+^/US^−^) at 3 days: (A) representative plots of the M1 macrophage marker CD86 and the M2 macrophage marker CD163, (B) the populations of F4/80^+^ CD86^+^ cells, and (C) the populations of F4/80^+^ CD163^+^ cells. (D–G) Relative genes mRNA expressions of RAW 264.7 cells exposed to different concentrations of BTO NPs with/without ultrasound irritation (US^+^/US^−^) at 3 days: the M1 macrophage‐related gene iNOs (D), and the M2 macrophage polarization‐related genes YM1 (E), Arginase‐1 (F), and Fizz1 (G). *: *p* < 0.05, **: *p* < 0.01 versus NC US^−^ group. ^: *p* < 0.05, ^^: *p* < 0.01 versus NC US^+^ group. #: *p* < 0.05, ##: *p* < 0.01 compared between different concentrations BTO group with/without ultrasound irritation (US^+^/US^−^).

We further conducted quantitative analysis using flow cytometry and real‐time RT‐PCR. From the result of flow cytometry analysis for detecting the expression of cell surface markers associated with macrophage polarization (Figure 3A–C), BTO NPs did not regulate the expression of the M1‐type marker CD86 (Figure 3B). In contrast, exposure to BTO NPs with US^+^ treatment substantially increased the expression of the M2‐type marker CD163 (from 9.33% to 13.08% with varying concentrations of BTO NPs), compared to the BTO NPs with US^−^ treatment group and the negative control (NC) group (Figure 3C). Moreover, real‐time RT‐PCR data revealed that the expression of M2 macrophage‐related genes, including YM1, Arginase‐1, and Fizz1, was strongly upregulated with increasing concentrations of BTO NPs under US^+^ treatment (Figure 3E–G). We observed a slight downregulation of M1 macrophages‐related genes with BTO NPs under US^+^ treatment, compared with BTO NPs under US^−^ treatment (Figure 3D). These results indicate that the piezoelectric stimulation under ultrasound irritation can modulate macrophage reprogramming towards the anti‐inflammatory M2 phenotype.^[^ ^15^ ^]^

### 2.3 Mechanism underlying piezoelectric stimulation mediated macrophage reprogramming

**FIGURE 4:**
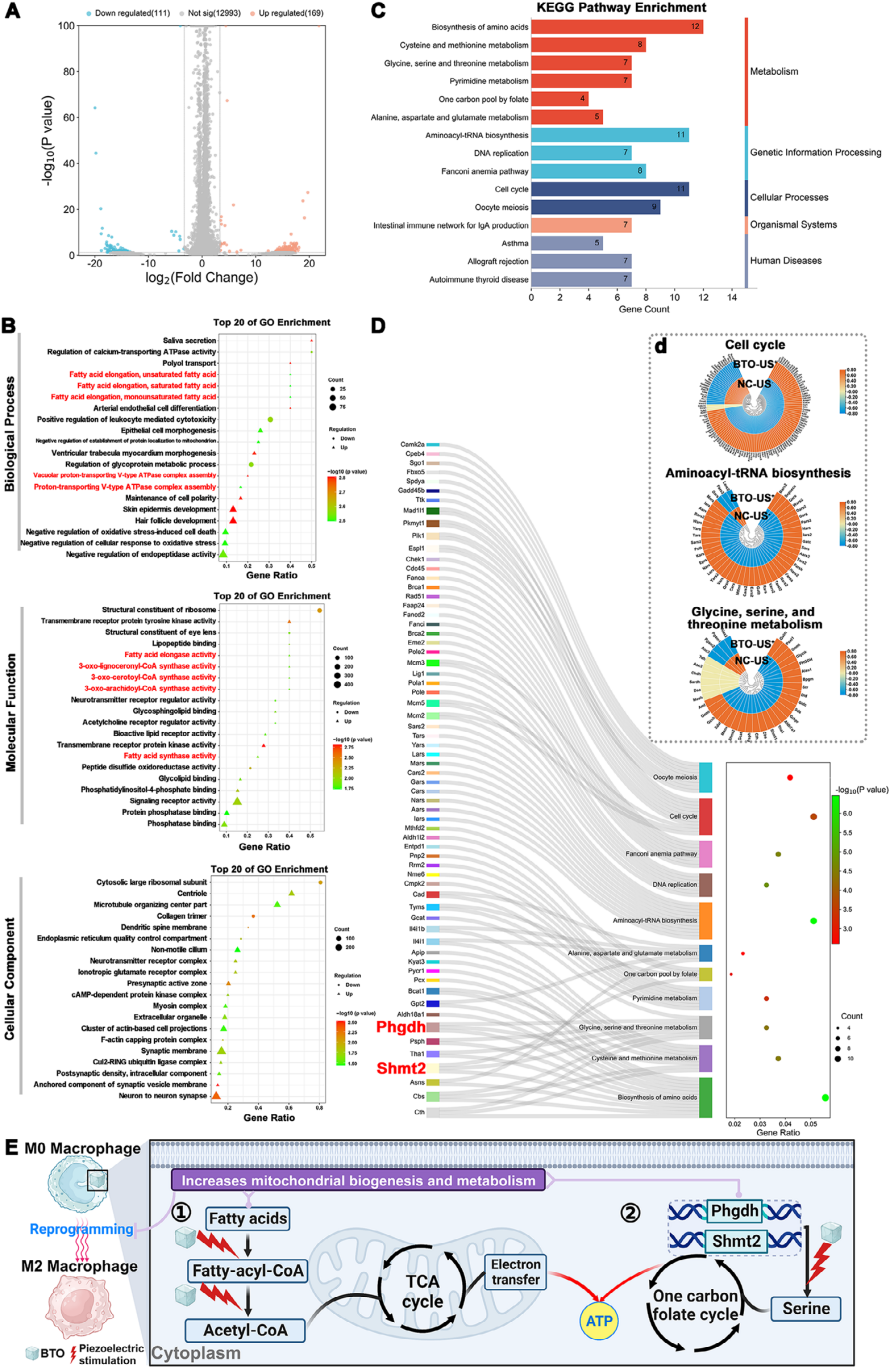
Differential gene expression profiles of RAW 264.7 cells between the NC US^−^ group and BTO US^+^ group. (A) Differentially expressed genes (DEGs) volcano plot. The *X*‐axis represents the multiple of the difference after the log2 conversion, and the *Y*‐axis represents the significance value after the −log10 conversion. Red denotes DEGs that are up‐regulated, blue denotes DEGs that are down‐regulated, and gray denotes non‐DEGs. (B) Top 20 bubble charts of the biological process, molecular function, and cellular component of GO enrichment analysis. (C) Classified KEGG pathways enrichment of the DEGs. The pathways were further classified into five major groups: metabolism, genetic information processing, cellular processes, organismal systems, and human diseases. (D) Sankey diagram of classified KEGG pathways enrichment involving metabolism (biosynthesis of amino acids; cysteine and methionine metabolism; glycine, serine, and threonine metabolism; pyrimidine metabolism; one carbon pool by folate; alanine, aspartate and glutamate metabolism) and genetic information processing (aminoacyl‐tRNA biosynthesis; DNA replication; Fanconi anemia pathway), and the heat map of differentially clustered for cell cycle, aminoacyl‐tRNA biosynthesis and glycine, serine, and threonine metabolism (inset). (E) The schematic representation of the piezoelectric effect‐mediated reprogramming of macrophages.

To gain a deeper understanding of the mechanism behind macrophage reprogramming mediated by piezoelectric stimulation, we performed RNA sequencing analysis on macrophages subjected to piezoelectric stimulation. As shown in Figure 4A, there were 280 differentially expressed genes (DEGs) identified in RAW 264.7 cells treated with BTO US^+^ compared to NC US^−^, in which 169 were up‐regulated while 111 were down‐regulated.

The gene ontology (GO) enrichment analysis revealed that the GO terms of piezoelectricity‐activated macrophages were primarily related to cellular metabolism modulation, including lipid and energy metabolism (Figure 4B). Lipid metabolism is indicated by the process of fatty acid elongation, fatty acid elongase activity, 3‐oxo‐arachidoyl‐CoA synthase activity, 3‐oxo‐cerotoyl‐CoA synthase activity, 3‐oxo‐lignoceronyl‐CoA synthase activity, and fatty acid synthase activity (Figure 4B, biological process and molecular function). Energy metabolism is indicated by the upregulation of genes involved in the process of “vacuolar proton‐transporting V‐type ATPase complex assembly” and “proton‐transporting V‐type ATPase complex assembly” (Figure 4B, biological process).

In general, M1 macrophages rely on glycolysis for energy production to carry out pro‐inflammatory function. In contrast, M2‐polorized macrophages exhibit an intact tricarboxylic acid cycle (TCA) with enhanced oxidative phosphorylation (OXPHOS) and increased expression of Arginase‐1 (Arg1) through fatty acid oxidation (FAO). The FAO pathway catalyzes arginine to urea and ornithine, facilitating collagen synthesis to promote wound healing.^[^ ^16^ ^]^ In addition, previous studies have reported that macrophage FAO is associated with reduced ROS production.^[^ ^17^ ^]^ Consistent with this finding, our RNA‐seq data demonstrated that piezoelectric stimulation results in protection against oxidative stress, as evidenced by the significant upregulation of DEGs involved in processes such as “negative regulation of oxidative stress‐induced cell death” and “negative regulation of cellular response to oxidative stress” (Figure 4B, biological process).

In addition, we conducted KEGG pathway‐enrichment analysis to further explore these DEGs (Figure 4C). The upregulated DEGs in the piezoelectricity‐stimulated macrophages were significantly enriched in pathways related to cell cycle, amino acid biosynthesis and energy metabolism, including aminoacyl‐tRNA biosynthesis, biosynthesis of amino acids, glycine/serine and threonine metabolism, cell cycle, cysteine and methionine metabolism and one carbon pool by folate. The network analysis of these pathways reviewed their convergence on aminoacyl‐tRNA biosynthesis, glycine, serine, and threonine metabolism, and the cell cycle (Figures S5, Supporting Information). As shown in Figure S5, aminoacyl‐tRNA biosynthesis, glycine, serine, and threonine metabolism, and the cell cycle are at the core of the functionally organized KEGG pathway network, with up to 15 pathways associated with glycine, serine, and threonine metabolism, and 9 pathways associated with aminoacyl‐tRNA biosynthesis. Notably, metabolic pathways such as the citrate cycle (TCA cycle), arginine and praline metabolism, cysteine and methionine metabolism, central carbon metabolism, phenylalanine, tyrosine, and tryptophan biosynthesis, and pyrimidine metabolism directly interacted with aminoacyl‐tRNA biosynthesis and glycine, serine, and threonine metabolism (Figures S5, Supporting Information), which are closely associated with M2 polarization of macrophages.^[^ ^18^ ^]^

To investigate the specific genes involved, we sketched the Sankey diagrams of KEGG pathway enrichment and heat maps displaying the heterogeneous clustering of genes related to the aforementioned pathways (Figure 4D and inset). Among these genes, several upregulated genes associated with glycine, serine, and threonine metabolism were identified, including Shmt2 (serine hydroxymethyltransferase 2), Phgdh (3‐phosphoglycerate dehydrogenase), Sars (seryl‐aminoacyl‐tRNA synthetase), and Gars (glycyl‐tRNA synthetase). Amino acid metabolism has been found to regulate the interconnected processes of glycolysis, TCA cycle, and OXPHOS, which collectively generate ATP.^[^ ^18a^ ^]^

Among these metabolic pathways, the serine synthesis pathway (SSP) plays a critical role in one‐carbon metabolism in various cell types.^[^ ^19^ ^]^ The entry step of the SSP from glycolysis in the cytoplasm to mitochondrial metabolism is catalyzed by the enzyme phosphoglycerate dehydrogenase (Phgdh), which produces serine.^[^ ^18a^ ^]^ Wilson et al. reported that Phgdh acts as a metabolic checkpoint in M2 macrophages, and its activation is critical for enhancing and maintaining the polarization of the M2 phenotype.^[^ ^19c^ ^]^ Additionally, mitochondrial translation initiation relies on one‐carbon (1‐C) unit generation and the modified tRNA formation from serine, facilitated by the catabolic enzyme serine hydroxymethyltransferase (Shmt2).^[^ ^18a^ ^]^

Interestingly, our RNA‐seq data also revealed a significant increase in the expression of both Phgdh and Shmt2 in the piezoelectricity‐activated macrophages compared with the negative control. Thus, it can be inferred that piezoelectric stimulation enhances fatty acid oxidation in macrophages to produce acetyl CoA, which enters the mitochondrial TCA cycle to generate electron donors for ATP production. Moreover, piezoelectric stimulation activates the serine synthesis pathway, leading to one‐carbon unit production through upregulation of Phgdh and Shmt2 expression, ultimately promoting mitochondrial biogenesis and metabolism (Figure 4E). Overall, these findings suggest that piezoelectric stimulation drives macrophage M2 polarization and metabolic reprogramming by enhancing amino acid biosynthesis and fatty acid oxidation to support mitochondrial biogenesis.

### 2.4 M2 macrophage polarization promoted osteogenic differentiation of BMSCs

**FIGURE 5:**
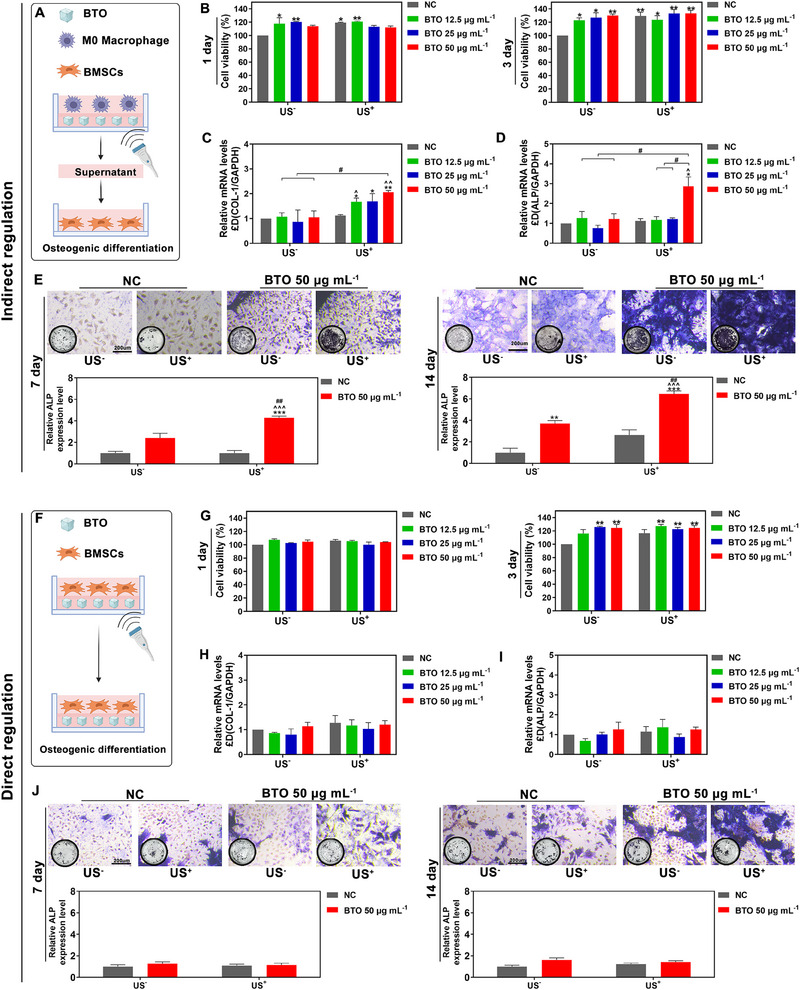
Osteogenic differentiation of BMSCs mediated by the cell‐free supernatants of piezoelectric stimulated (A–E) macrophages and (F–J) BTO NPs in vitro. (A) Schematic illustration for the indirect regulation of BMSCs. (B) Cell viability of BMSCs exposed to the cell‐free supernatant from the RAW 264.7 cells treated with the different concentrations of BTO with/without ultrasound irritation (US^+^/US^−^). (C,D) The gene expression of COL‐1 and ALP in BMSCs exposed to the cell‐free supernatant from the RAW 264.7 cells treated with the different concentrations of BTO with/without ultrasound irritation (US^+^/US^−^). (E) ALP staining and relative ALP level of BMSCs exposed to the cell‐free supernatant from the RAW264.7 cells treated with cell medium or 50 µg mL^−1^ BTO with/without ultrasound irritation (US^+^/US^−^). (F) Schematic illustration for the direct regulation of BMSCs. (G) Cell viability of BMSCs exposed to the different concentrations of BTO with/without ultrasound irritation (US^+^/US^−^). (H,I) the gene expression of COL‐1 and ALP in BMSCs exposed to the different concentrations of BTO with/without ultrasound irritation (US^+^/US^−^). (J) ALP staining and relative ALP level of BMSCs exposed to cell medium or 50 µg mL^−1^ BTO with/without ultrasound irritation (US^+^/US^−^) *: *p* < 0.05, **: *p* < 0.01, ***: *p* < 0.001 versus NC US^−^ group. ^: *p* < 0.05, ^^: *p* < 0.01, ^^^: *p* < 0.001 versus NC US^+^ group. #: *p* < 0.05, ##: *p* < 0.01 compared between the different concentrations of BTO with/without ultrasound irritation (US^+^/US^−^).

Previous studies have indicated that phenotype macrophages can induce osteogenic differentiation of BMSCs through the secretion of cytokines such as IL‐10 and IL‐4.^[^ ^20^ ^]^ To investigate whether piezoelectric stimulation can effectively promote osteogenesis of BMSCs through M2 polarization of macrophages, RAW 264.7 cells were incubated with BTO NPs and subjected to US^+^ treatment to simulate a mechanic microenvironment similar to load‐bearing bone. After 3 days of co‐incubation, the cell‐free supernatants from piezoelectrically stimulated macrophages were collected to treat BMSCs (Figure 5A). As a result, the cell viability of BMSCs was not suppressed by the supernatants obtained from macrophages treated with different concentrations of BTO with US^+^ treatment (Figure 5B). The expression of osteogenic differentiation markers of BMSCs (COL‐1 and ALP) were significantly upregulated in a dose‐dependent manner. Particularly, at a concentration of 50 µg mL^−1^, BTO NPs combined with US^+^ treatment significantly increased the gene expression levels of COL‐1 (≈2.1‐fold) and ALP (≈2.9‐fold) compared to the NC US^−^ group (Figure 5C,D). In contrast, the supernatants of macrophages from the BTO NPs with US^−^ treatment did not increase the gene expression of ALP and COL‐1 in BMSCs. The expression, synthesis, and secretion of ALP and COL‐1 are well‐known early markers of osteogenesis. ALP contributes to bone mineralization by hydrolyzing inorganic pyrophosphate and supplying inorganic phosphate for hydroxyapatite formation.^[^ ^21^ ^]^ The dynamic assembly of bone collagen, particularly COL‐1, is essential for the encapsulation and mineralization of osteocytes within the bone matrix.^[^ ^22^ ^]^ Therefore, ALP staining was conducted to observe the mineralization of BMSCs. At 7 and 14 days, the ALP results demonstrated a considerable amount of blue‐purple formazan in BMSCs treated with the supernatants of piezoelectrically stimulated macrophages, indicating a higher degree of mineralization compared to the other control groups (Figure 5E).

In addition, we also investigated whether the piezoelectric BTO NPs could also directly induce osteogenic differentiation of BMSCs (Figure 5F). Our results revealed that when BMSCs were treated with 12.5–50 µg mL^−1^ BTO NPs combined with US^+^ treatment, although the cell viability of BMSCs was not significantly suppressed (Figure 5G), there was no significant change in ALP and COL‐1 expression (Figure 5H,I). Additionally, ALP staining did not show any noticeable differences between the groups (Figure 5J). Collectively, our finding demonstrated that piezoelectric stimulation can effectively reprogram macrophages into an M2‐phenotype, which subsequently induced osteogenesis.

### 2.5 In vivo regeneration of alveolar bone defects by BTO‐embedded composite hydrogel

**FIGURE 6:**
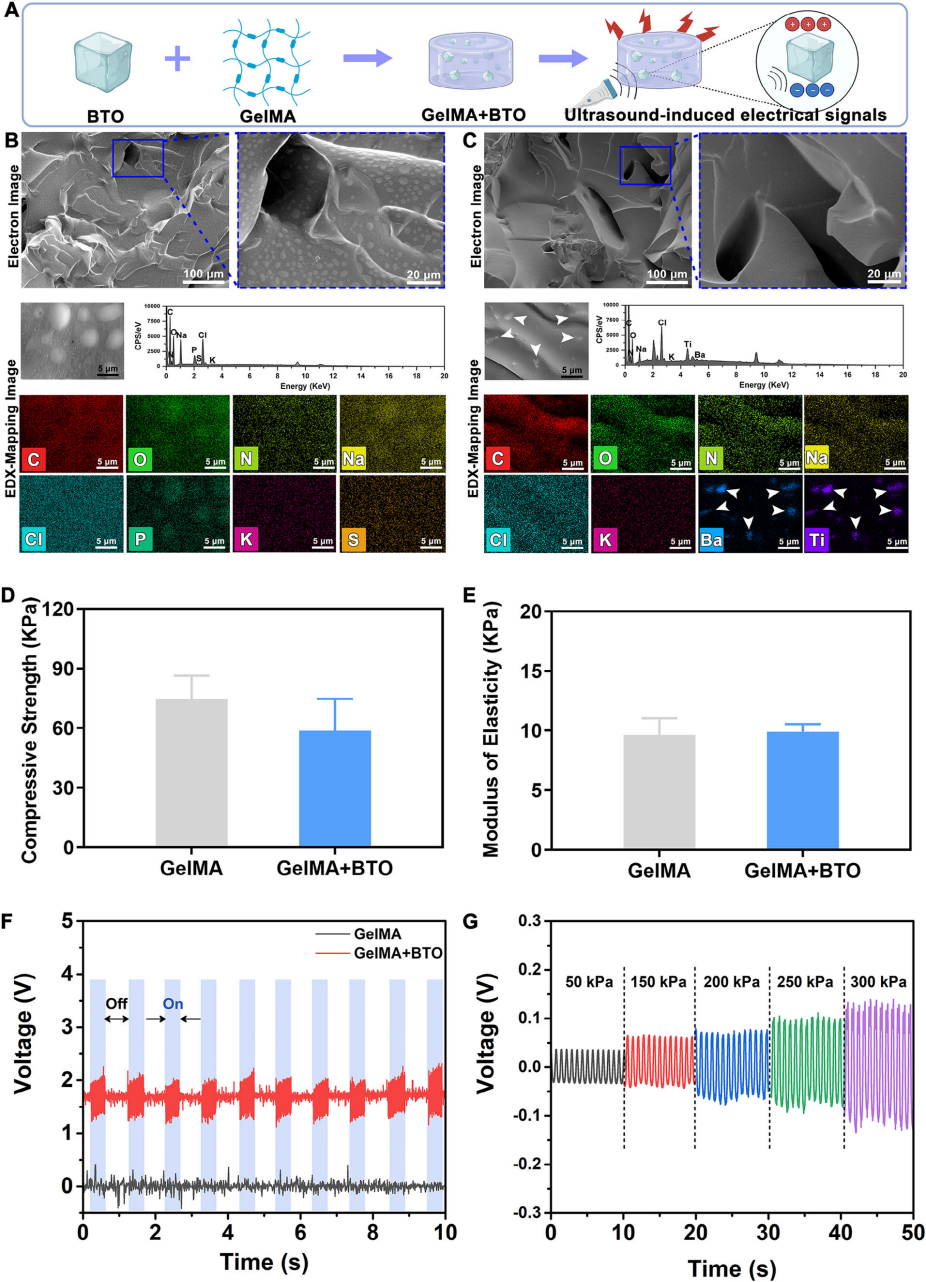
Characterization of BTO‐embedded nanocomposite hydrogel. (A) Schematic representation for the construction of BTO‐embedded nanocomposite hydrogel. (B,C) SEM images and EDX surface‐scan element distribution of GelMA and GelMA+BTO hydrogel. White arrows indicated BTO NPs. (D) Compressive strength and (E) elastic modulus of GelMA and GelMA+BTO hydrogel. (F) Mechanical‐electric response of GelMA and GelMA+BTO hydrogel under on/off ultrasonic stimulation. (G) Open‐circuit voltages of devices made from GelMA+BTO hydrogel under variable forces from 50 to 300 kPa.

To assess the potential of piezoelectric stimulation for bone regeneration, we conducted in vivo experiments using a rat mandibular alveolar bone defect model.^[^ ^4^ ^]^ To ensure the retention of BTO NPs at the site of bone defects, we incorporated them into GelMA hydrogel to fabricate BTO‐embedded nanocomposite hydrogel (Figure 6A). No significant particulate substances consisting of Ba and Ti elements were observed in GelMA alone (Figure 6B); in contrast, SEM‐EDX mapping showed a scattered distribution of nanoparticles in the hydrogel and displayed significant Ti and Ba elements (Figure 6C), indicating the effective incorporation of BTO NPs into the GelMA hydrogel (GelMA+BTO hydrogel). The compressive strengths of GelMA and GelMA+BTO hydrogels ranged from 42 to 88 kPa (Figure 6D), and the corresponding elastic moduli were 9.64 ± 1.40 kPa and 9.91 ± 0.63 kPa (Figure 6E), respectively, suggesting that the incorporation of BTO NPs does not significantly alter the mechanical properties of the hydrogel.

Next, we investigated whether BTO NP incorporation conferred piezoelectric property to the composite hydrogels. As shown in Figure 6F, when assembled into a piezoelectric generator, GelMA hydrogels alone did not exhibit any ultrasound‐induced electrical signals, while the BTO‐embedded composite hydrogel generated stable voltages of approximately 2 V when exposed to US, indicating that the BTO‐embedded hydrogels can generate piezoelectric stimulation in response to mechanical force. We observed that the output voltages increased gradually with higher mechanical stress ranging from 50 to 300 kPa (Figure 6G). Furthermore, in vitro degradation experiments revealed that the composite hydrogels exhibited sustained release of BTO NPs up to 8 weeks (Figures S6, Supporting Information), which provides a basis for the long‐term piezoelectric stimulation in vivo.

**FIGURE 7:**
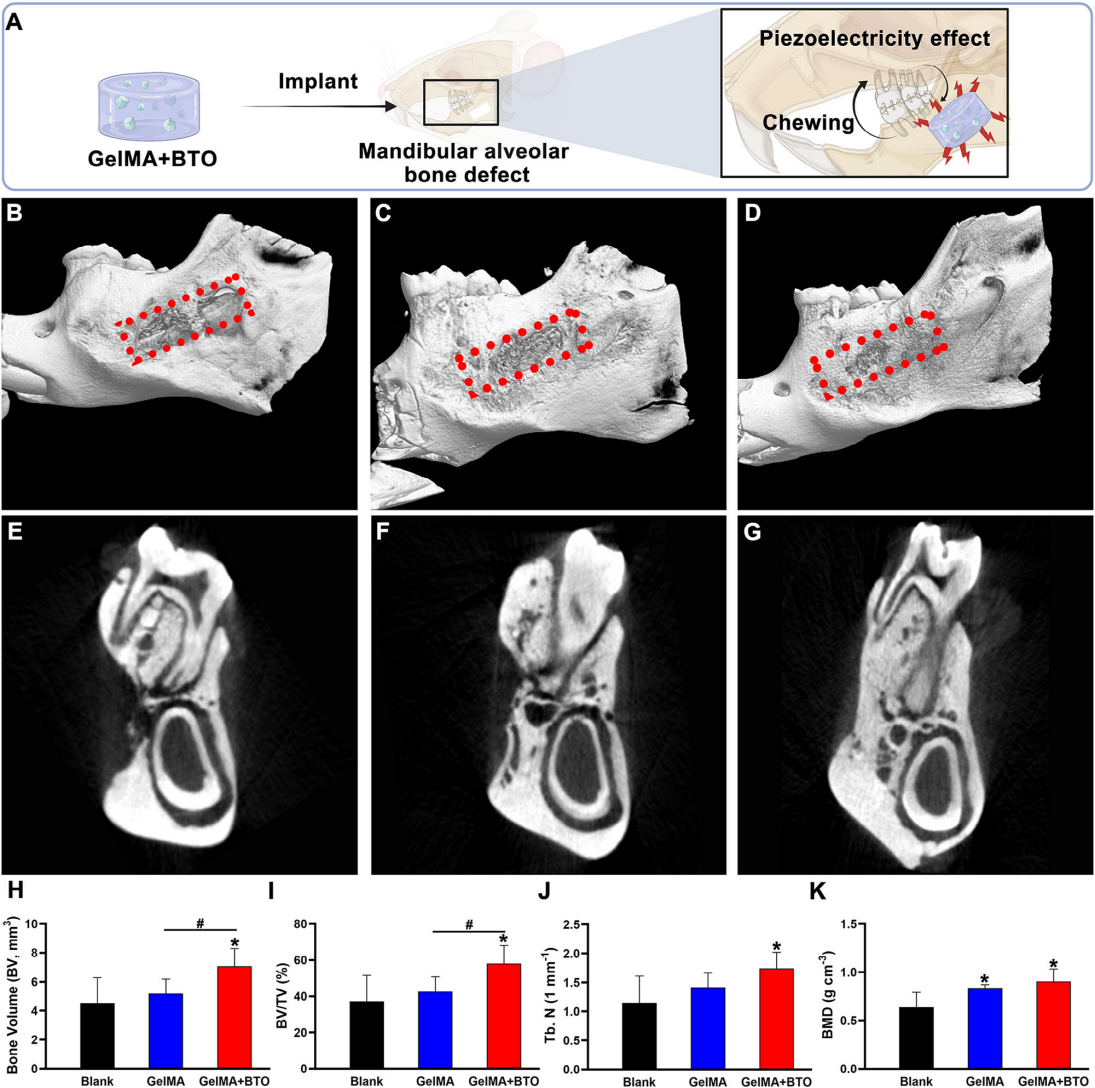
Mandibular alveolar regeneration mediated by BTO‐embedded nanocomposite hydrogel. (A) Schematic diagram of a mandibular alveolar bone defect model and piezoelectric effect generated by BTO‐embedded nanocomposite hydrogel. (B–G) Representative micro‐CT images for each group at 8 weeks: (B,E) Blank group, (C,F) GelMA group and (D,G) GelMA+BTO group. The red dashed box represents the initial defect area. (H) Quantitative analysis of BV, (I) BV/TV, (J) Tb.N, and (K) BMD in the defect area at 8 weeks. BV: new bone volume, BV/TV: trabecular bone volume percentage, Tb.N: trabecular number, BMD: bone mineral density. *: *p* < 0.05 versus blank group, #: *p* < 0.05 compared between GelMA and GelMA+BTO group. *n* = 5 for each group.

Further, we assessed the regeneration of mandibular alveolar bone defects following 8 weeks local injection of the BTO‐embedded composite hydrogel (Figure 7A). Micro‐computed tomography (Micro‐CT) analysis revealed that the mandibular alveolar bone defect region in the GelMA+BTO group was almost completely filled with newly formed bone tissues, whereas the GelMA and the blank group showed limited regeneration of new bone around the defect boundaries (Figure 7B–G). Quantitative analysis demonstrated that the GelMA+BTO group exhibited the highest bone volume (BV) values, and significantly increased the trabecular bone volume percentage, trabecular number (Tb.N), and bone mineral density (BMD) compared to the GelMA and blank groups (Figure 7H–K).

**FIGURE 8:**
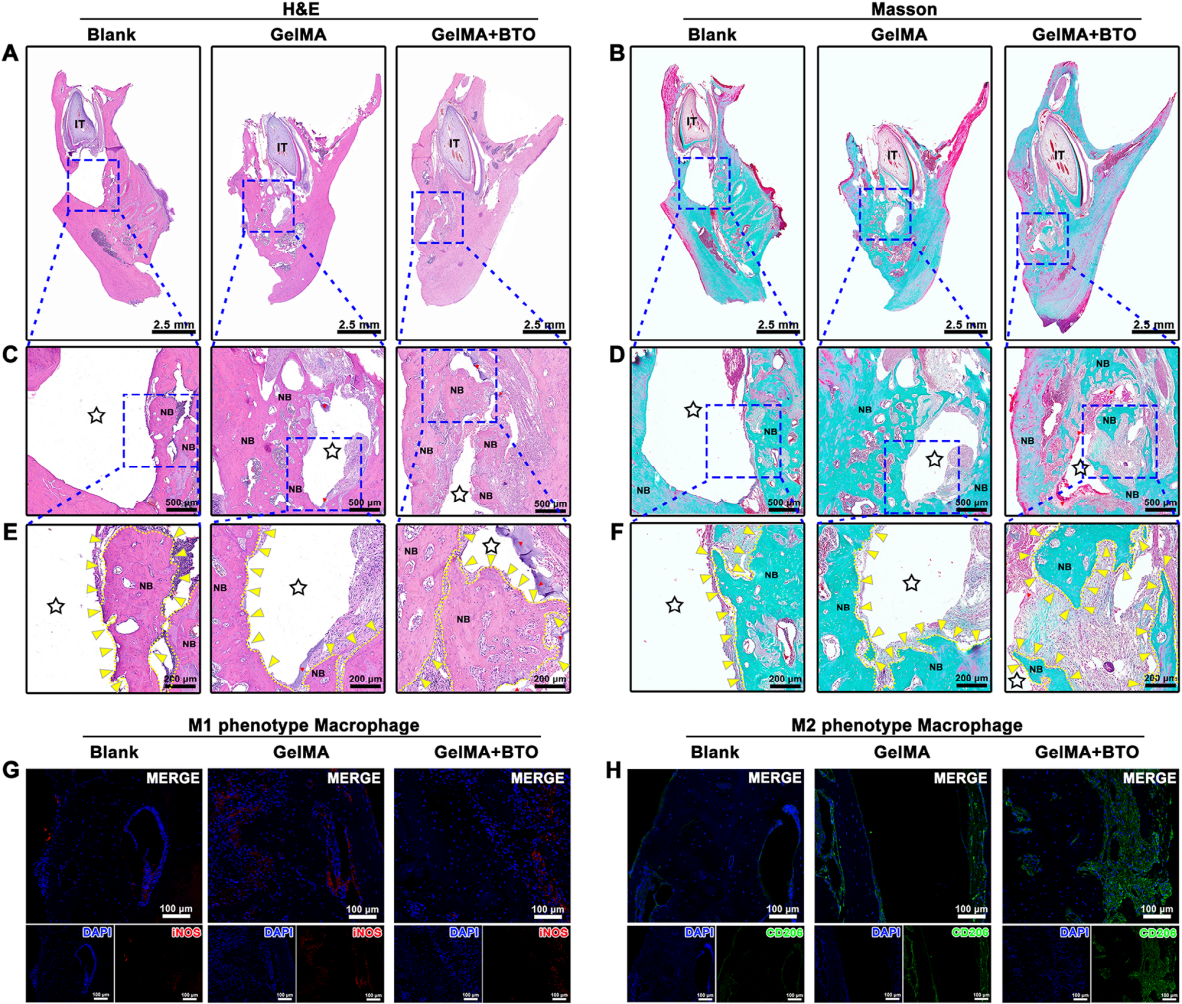
Histological and immunofluorescence staining of mandibular alveolar bone regeneration mediated by BTO‐embedded nanocomposite hydrogel. (A,C,E) H&E staining of and (B,D,F) Masson's trichrome staining of the mandibular alveolar defect after the implantation at 8 weeks: (A,B) the full‐view slide of the mandibular alveolar defect defects, (C,D) the 4× magnification of the blue dashed boxes in (A, B), (E,F) the 10× magnification of the blue dashed boxes in (A, B). The star represents the area of the mandibular alveolar defect. IT: incisor tooth, NB: new bone. (G,H) Immunofluorescence staining of macrophage polarization in the mandibular alveolar defect after the implantation at 8 weeks: (G) the immunostaining of M1 phenotype macrophage marker iNOs (red), (H) the immunostaining of M2 phenotype macrophage marker CD206 (green).

Furthermore, in the GelMA+BTO group, histological analyses including H&E and Masson staining demonstrated a robust growth of newly formed bone tissue from the periphery towards the center of the defect site, leading to near‐complete healing of the defect area (Figure 8A–F). Additionally, the surrounding new bone tissue exhibited a mature lamellar type, while the new bone in the center of the defect showed characteristics of woven bone, with osteocytes randomly embedded within lacunae. Masson staining also revealed the presence of filamentous collagen, stained light blue, between the margins of the newly formed bone (Figure 8F). In contrast, the GelMA and the blank groups only displayed limited amounts of mature lamellar bone formation around the defect boundaries (Figure 8).

Consistent with our in vitro findings, our in vivo results provide unequivocal evidence that the BTO‐embedded hydrogels promoted collagen synthesis and mineralization within the bone defect region, ultimately leading to endogenous bone regeneration. The piezoelectric stimulation under a mechanic microenvironment is believed to play a crucial role in this process. Previous studies have reported that electrical stimulation through implanted electrodes derived from the aortic sinus nerve can reduce alveolar bone loss in periodontal disease.^[^ ^23^ ^]^ In our study, BTO NPs are capable of generating piezoelectrical stimulation when subjected to wireless mechanical stimuli. When the BTO embedded hydrogels were implanted into mandibular alveolar bone defects located in the posterior area of the lateral mandible, they experience load‐bearing forces during chewing and gnawing,^[^ ^24^ ^]^ therefore producing piezoelectric stimulation. This piezoelectric stimulation facilitates endogenous bone regeneration of mandibular alveolar bone defects within a mechanical microenvironment.

To further confirm the role of macrophage polarization in piezoelectric stimulation mediated bone regeneration, immunofluorescent staining was performed to assess the expression of M1 and M2 phenotypes in the repaired bone tissue region. Immunofluorescent staining results (Figure 8G,H) revealed low accumulation of M1 macrophages (iNOS‐positive, shown in red) in all three groups. However, the number of M2 macrophages (CD206‐positive, shown in green) was considerably higher in the GelMA+BTO group compared to the GelMA group. In addition, during the 8 weeks of implantation, the BTO‐embedded composite hydrogel displayed a promising biosafety according to the hematological, biochemical, and histological results (Figure S7, Supporting Information). The piezoelectric stimulation mediated by BTO NPs in response to mechanic microenvironment can effectively promote bone healing in alveolar bone defects at load‐bearing sites through modulation of M2 macrophage polarization in vivo.^[^ ^25^ ^]^ All these findings indicate that the integrated BTO NPs and hydrogel to construct a piezoelectric hydrogel, which ensures the dual advantages of stable electrical signal output and flexibility in filling, providing an important advantage for endogenous electric field modulation of macrophage phenotypes and triggering tissue regeneration.

## 3 CONCLUSION

In conclusion, we investigated the osteoimmunomodulatroy effects of piezoelectric stimulation within a mechanical microenvironment. Piezoelectric stimulation efficiently induced macrophage reprogramming towards the pro‐regenerative/anti‐inflammatory M2 phenotype. The polarization of macrophages subsequently contributed to the induced osteogenic differentiation of BMSCs. Through RNA‐seq analysis, we also identified a close association between piezoelectric stimulation‐mediated macrophage M2 polarization and metabolic reprogramming, including enhanced amino acid biosynthesis, fatty acid oxidation, and glycolysis switching. Additionally, we developed a piezoelectric composite hydrogel for promoting bone regeneration in a rat mandibular alveolar bone defect model, through remodeling an osteo‐friendly immune microenvironment via M2 macrophage polarization. The piezoelectricity‐driven osteoimmunomodulation proposed in this study provides new insights into the design of next‐generation bioactive materials with combined osteoinductive, osteoimmunomodulatory and piezoelectric activity.

## 4 EXPERIMENTAL SECTION

### 4.1 Synthesis of BTO NPs

The piezoelectric BaTiO_3_ nanoparticles (BTO NPs) was synthesized through a solvothermal and calcination process.^[^ ^26^ ^]^ Briefly, 50 mmol of Ti[O(CH_2_)_3_CH_3_]_4_ (Ti‐butoxide, 97%, Macklin) was mixed with 20 mL of ethanol and supplemented with 7 mL of ammonium hydroxide solution (NH_3_·H_2_O, 25%, Sinopharm Chemical Reagent Co., China). Meanwhile, 75 mmol of Ba (OH)_2_·8H_2_O (Ba‐hydroxide, 98%, Aladdin, China) was dissolved in 25 mL of deionized water under constant agitation until a clear solution formed. The aqueous Ba‐hydroxide solution was added to the Ti precursor mixture under gentle stirring. To control the particle size and morphology, triethanolamine (TEA, 98%, Sinopharm Chemical Reagent Co., China) was added to the mixed solution. The resulting suspension was transferred to an autoclave and heated at 200°C for 48 h. For thermal calcination, the BTO NPs was placed in a corundum crucible and the temperature was increased to 800°C at a heating rate for 2°C min^−1^ for 4 h. Finally, the products were naturally cooled and ground in a mortar for further use.

### 4.2 Characterizations of BTO NPs

X‐ray diffraction (XRD) spectra were acquired on a D/MAX‐TTRIII XRD system (Japan) equipped with Cu Kα radiation. Scanning electron microscopic (SEM) images were obtained on a field‐emission S‐4800 microscope (Hitachi, Japan). Transmission electron microscopic (TEM) photographs were captured using a Tecnai G2 F20 high‐resolution electron microscopy (FEI Company, US). Energy‐dispersive X‐ray (EDX) mapping was performed using high‐angle annular dark‐field (HAADF) scanning transmission electron microscopy (STEM). Raman spectra were collected using a laser Raman spectrometer (LABRAM HR EVOLUTION, HORIBA JY, France) at an excitation wavelength 532 nm. Piezoresponse force microscopic (PFM) measurements and amplitude‐butterfly loop of BTO NPs are conducted using an atomic force microscope (MFP‐3D, Asylum Research) in PFM mode. Polarization‐electric field hysteresis loops were measured using a ferroelectric tester (RTI‐MultiFerroic, Radiant, USA).

### 4.3 Finite element modeling (FEM) simulation of BTO NPs

A multiphysics field simulation was implemented to study the dynamic piezoelectricity of 3D BTO NPs.^[^ ^27^ ^]^ This model employed cubic‐shaped BTO nanocrystals with a side length of 145 nm. The polarization was oriented along the *z*‐axis of the global coordinate system, with the center fixed and grounded. The material parameters required for the simulation, including density (*ρ*), elasticity matrix (*cE*), coupling matrix (*eES*), and relative permittivity (*ε_r_*) of the BTO NPs, were set as predefined material parameters in COMSOL Multiphysics software. Under the ultrasound wave with a frequency of 1 MHz and a power of 1 W cm^−1^, the pressure force was calculated to be 10^6 ^Pa.

### 4.4 In vitro studies

#### 4.4.1 Cell culture and viability assay

A mouse macrophage cell line (RAW 264.7) and mouse bone marrow‐derived mesenchymal stem cells (BMSCs) were utilized in this study. RAW 264.7 cells were acquired from the Chinese Academy of Sciences Cell Bank (Shanghai, China). All the animal experiments were ethically reviewed and approved by the Independent Ethics Committee of Shanghai Ninth People's Hospital, Shanghai Jiao Tong University School of Medicine (Ethical number: SH9H‐2022‐A417‐SB). Standard protocols were followed for the extraction of BMSCs.^[^ ^28^ ^]^ Briefly, bone marrow cells were extracted from C57BL/6 mice (6–8 weeks old) by flushing the femurs and tibias. The cells were then suspended in L‐DMEM (Hyclone, USA) supplemented with 10% MSC‐qualified FBS (Gibco, USA), 100U mL^−1^ penicillin, and 100 µg mL^−1^ streptomycin (Hyclone, USA). The culture medium was refreshed on day 3 to remove non‐adherent cells, and BMSCs from the second to fourth passages were used for the subsequent experiments.

The RAW 264.7 cells were seeded in 96‐well plates and allowed to adhere for 24 h before treatment with different concentrations of BTO (50, 100, 500, and 1000 µg mL^−1^) to assess its impact on cell viability. After incubation for 24 h, the viability of the cells was evaluated using the CellTiter 96 AQueous One Solution Cell Proliferation Assay (MTS, Promega, USA).

#### 4.4.2 Modulation of macrophage phenotype by BTO NPs

##### Cell treatment

RAW 264.7 cells were seeded at a density of 1 × 10^5^ cells per well in 24‐well plates. Three different doses of BTO NPs (12.5, 25, and 50 µg mL cells well^−1^) were used to stimulate the RAW 264.7 cells, without ultrasound (US^−^) or with ultrasound (US^+^) irritation. The ultrasound parameters were set as follows: 1.0 W cm^−2^, 1 MHz, 50% duty cycle, and 1‐minute duration. As a negative control (NC), the cells were treated only with the culture medium.

##### Macrophage morphology observation

The morphology of RAW 264.7 cells was assessed using filamentous actin (F‐actin) staining after 3‐days of piezoelectric stimulation with BTO NPs. To visualize the cells, RAW 264.7 cells were stained with Phalloidin‐iFluorTM 488 Conjugate (Cayman Chemical, USA) and DAPI (Solarbio, China) in darkness. A stained RAW 264.7 cells were then examined using a cell imaging multi‐mode reader (Cytation 3, BioTek Instruments, USA). Furthermore, fixed and dehydrated RAW 264.7 cells were subjected to SEM and TEM observation.

##### Flow cytometry and quantitative real‐time PCR (qRT‐PCR) analysis

Three days after BTO NPs treatment, flow cytometry and qRT‐PCR tests were utilized to identify macrophage phenotype‐related markers and genes. After being rinsed with PBS, RAW 264.7 cells were fixed and permeated using the Fixation/Permeabilization kit (BD Cytofix/CytopermTM, USA). Subsequently, the cells were stained with PE anti‐mouse F4/80 (BD Horizon TM, USA), BV421 anti‐mouse CD86 (a cell surface marker for M1 macrophage, BD Horizon TM, USA), and BV605 anti‐mouse CD163 (a cell surface marker for M2 macrophage, BD Horizon TM, USA) in the dark at room temperature. The expression of cell surface markers was assessed using a flow cytometer (FACSCelesta, BD, USA).

Additionally, total RNA was extracted from RAW 264.7 cells using the RNeasy Mini Kit (QiAGEN, Germany) for subsequent qRT‐PCR analysis. The PrimeScript RT reagent Kit (Takara, Japan) was used to synthesize complementary DNA (cDNA) from the extracted total RNA. qRT‐PCR was performed using the SYBR Premix EX Taq (Takara, Japan) and a detection system (Light cycler96, Roche, Switzerland). The primer sequences used are listed in Table S1.

##### Sequencing and data processing

The RNA of RAW 264.7 cells in the NC US^−^ and 50 µg mL^−1^ BTO US^+^ group at 3 days post‐treatment was extracted using TRIzol Reagent (ThermoFisher, USA). A cDNA library was constructed for subsequent data processing. The purified cDNA underwent end‐repair and PCR amplification. The insertion fragments of the cDNA libraries were analyzed using the Agilent 2100 Bioanalyzer, the effective concentration of the cDNA libraries was determined using the ABI StepOnePlus Real‐Time PCR System. After filtering out low‐quality reads, the remaining clean reads were aligned to the reference genome using Hierarchical Indexing for Spliced Alignment of Transcripts (HISAT).^[^ ^29^ ^]^ Bowtie2 was employed to align the clean reads to the reference gene sequence.^[^ ^30^ ^]^

To quantify gene expression levels in each sample, we utilized RSEM, a software tool for RNA‐seq analysis that evaluates computational gene reading and transcript isoform expression levels. Differential expression analysis was performed using the R software with statistical significance defined as Padj < 0.05 and foldchange > 2. The functional enrichment analysis of the resulting gene cluster was carried out using the Gene Ontology (GO) database. Furthermore, to explore the primary biological functions, metabolic pathways, and regulatory networks impacted by changes in macrophage polarization, the Kyoto Encyclopedia of Genes and Genomes (KEGG) pathway enrichment analysis of differentially expressed genes was conducted.

#### 4.4.3 Regulation of BMSCs differentiation by BTO NPs

##### Treatment of BMSCs with BTO NPs‐induced RAW 264.7 conditioned supernatants

The conditioned supernatants were obtained by treating RAW 264.7 cells as previously described, resulting in the collection of 8 groups of supernatants: (i) NC US^−^, (ii) 12.5 µg mL^−1^ BTO US^−^, (iii) 25 µg mL^−1^ BTO US^−^, (iv) 50 µg mL^−1^ BTO US^−^, (v) NC US^+^, (vi) 12.5 µg mL^−1^ BTO US^+^, (vii) 25 µg mL^−1^ BTO US^+^, and (viii) 50 µg mL^−1^ BTO US^+^. For subsequent assays, BMSCs were cultured in medium supplemented with the eight groups of supernatants at a 1:1 ratio, respectively. BMSCs were seeded in a 96‐well plate at a density of 3 × 10^3^ cells per well for the cell viability experiment and cultured in the 8 groups of supernatants for 1 and 3 days, respectively. Afterward, MTS was added to each well to measure the absorbance. PCR analysis of BMSCs was performed after seeding cells at a density of 1 × 10^4^ cells per well in 24‐well plates. After 24 h, cell cultures were replaced, and BMSCs were incubated with medium for 5 days, with supernatants changed every two days. Total RNA was extracted from MSCs, followed by cDNA reverse transcription, and expression of osteogenic differentiation genes ALP and COL‐1 was detected by PCR. To normalize the data, GAPDH was used as a housekeeping gene. Table S2 contains the primer sequences utilized for the osteogenic differentiation analysis of BMSCs.

ALP staining was conducted to identify the osteogenic differentiation. BMSCs were seeded in 24‐well plates at a density of 1 × 10^4^ cells per well for a 7‐day culture and a density of 5 × 10^3^ cells per well for a 14‐day culture. After incubation for 7 and 14 days, ALP staining was performed using the BCIP/NBT ALP kit (Beyotime, Shanghai, China).

##### Treatment of BMSCs with BTO NPs

To investigate the immediate modulatory effects of piezoelectric BTO NPs on BMSC osteogenic differentiation, MSCs were cultured with varying consternations of BTO NPs (12.5, 25, and 50 µg mL^−1^) without ultrasound (US^−^) or with ultrasound (US^+^). The negative control (NC) group was treated with culture medium. Subsequently, cell viability, expression of osteogenic differentiation‐related genes, and ALP staining were assessed and evaluated for direct induction of BMSC by BTO NPs.

### 4.5 Preparation and characterization of BTO‐embedded composite hydrogels

To further validate the effectiveness of piezoelectric stimulation on bone regeneration in vivo, a piezoelectric nanocomposite hydrogel loaded with BTO NPs was fabricated. The lyophilized gelatin methacryloyl (GelMA, EFL‐GM‐90) and photoinitiator lithiumphenyl‐2,4,6‐trimethylbenzoylphosphinate (LAP) were obtained from EFL (EngineeringforLife Group, China). BTO‐based composite hydrogels were prepared by dispersing 500 µg BTO in 1 mL of 10% w/v GelMA in PBS solution with 0.25% w/v LAP, and exposing it to a light source (405 nm) for 30 s. The resulting composite hydrogel sample was designed as the GelMA+BTO group, while a 10% w/v GelMA hydrogel sample served as the non‐piezoelectric control group (GelMA group).

SEM‐EDX mapping (Mira3, Tescan, Czechia) was employed to observe and analyze the morphology of the samples and the distribution of nanoparticles in the composite hydrogels. Cylindrical BTO‐based composite hydrogels (ø 4 × 6 mm) were prepared for mechanical property testing and measured using universal material testing equipment. The piezoelectric properties of BTO‐embedded composite hydrogels under different pressures, with or without US stimulation, were determined using a piezoelectric test system consisting of a universal digital meter (Keithley 6514, USA). To evaluate the leachability of BTO NPs from the composite hydrogel, 100 µL of cured BTO‐based hydrogel was immersed in 1 mL of PBS at 37°C for 8 weeks, and the leached BTO NPs were quantified using ICP‐MS (Agilent 7700s, USA).

### 4.6 In vivo studies

#### 4.6.1 Animal surgical procedure

Fifteen healthy 8‐week‐old male Sprague–Dawley (SD) rats weighing between 220 and 250 g were randomly divided into three groups: Blank, GelMA, and GelMA+BTO group. All surgical procedures were performed under general anesthesia induced by a 3% pentobarbital sodium intraperitoneal injection. The rat maxillofacial bone defect model was established.^[^ ^31^ ^]^ Briefly, a 1‐cm‐long skin incision was made approximately 2 mm from the left side of each rat, parallel to the angle formed by the mouth‐ear line. The masseter muscles and periosteum covering the buccal surface of the mandible were carefully separated from the bone to expose the mandible. Using a low‐speed dental handpiece, a mandibular defect of approximately 5 × 2 × 2 mm was created in the posterior region of the lateral mandibular, parallel to the top of the alveolar ridge, rinsed with saline to cool it down during the procedure. Gauze was used to aspirate the bleeding within the defect. In the GelMA+BTO group, the mandibular defect was injected with BTO‐embedded composite hydrogels and subsequently subjected to ultraviolet light (405 nm) curing for 30 s. The sham operation served as the blank group, while the GelMA hydrogel injected into the mandibular defect represented the GelMA group.

#### 4.6.2 Micro‐CT imaging

Eight weeks after surgery, mandible samples were collected and subjected to micro‐CT scanning and analysis (Skyscan 1076, Bruker, USA). The CT scanning parameters were set at 40 kV and 250 µA, with a camera pixel size of 12.60 µm. 3D image reconstruction and quantitative data analysis, including measurements of new bone volume (BV), trabecular bone volume percentage (BV/TV), trabecular number (Tb. N), and bone mineral density (BMD) were performed using the CT‐Analyzer software (Bruker, USA).

#### 4.6.3 H&E, Masson, and immunofluorescence were stained for the mandibular defect area

After micro‐CT examination, the mandible samples were decalcified in an EDTA solution and embedded in paraffin. Hematoxylin and eosin (H&E) staining and Masson's trichrome staining were performed on mandible slices to visualize new bone formation and residual scaffold. To identify the macrophage phenotype within the rat mandibular defects, immunofluorescence staining was conducted using monoclonal antibodies of iNOS (Abcam, UK) as an M1 marker and CD206 (Abcam, UK) as an M2 marker.

#### 4.6.4 Subchronic toxicity assessment

To evaluate the systemic toxicity of BTO‐embedded composite hydrogel, blood and major organs of the rats were collected alongside the implantation experiments. Blood samples were collected for hematological and blood biochemical analyses, including white blood cells (WBC), red blood cells (RBC), platelets (PLT), granulocytes, lymphocytes, monocytes, alanine aminotransferase (ALT), aspartate aminotransferase (AST), lactic dehydrogenase (LDH), alkaline phosphatase (ALP), creatinine (CREA), urea nitrogen (UREA), uric acid (UA), total cholesterol (TC), and triacylglycerol (TG). The heart, lung, liver, spleen, and kidney were harvested for H&E staining.

### 4.7 Statistical analysis

The data are presented as the mean ± standard deviation. SPSS 20.0 software was used for statistical analysis. The statistical comparisons were established using the *t*‐test. The significant differences (*p*‐value < 0.05) were identified by one‐way analysis of variance (ANOVA).

## CONFLICT OF INTEREST STATEMENT

The authors declare no conflicts of interest. Linlin Li is a member of the *Exploration* editorial board, and she was not involved in the handling or peer review process of this manuscript.

## Supporting information

Supporting information

## Data Availability

The data that support the findings of this study are available from the corresponding author upon reasonable request.
