# Supplementary material for: Piezoelectric stimulation enhances bone regeneration in alveolar bone defects through metabolic reprogramming of macrophages
Source: Exploration (Beijing). 2024 Jun 10;4(6):20230149. doi: 10.1002/EXP.20230149 (PMC11657998; doi:10.1002/EXP.20230149)
Supplement: Supplementary file 1 — Supporting information [file EXP2-4-20230149-s001.docx]

Supporting Information

Piezoelectric Stimulation Enhances Bone Regeneration in Alveolar Bone Defects through Metabolic Reprogramming of Macrophages

*Baiyan Sui ^1^, Tingting Ding ^1^, Xingyi Wan ^2, 3^, Yuxiao Chen ^1^, Xiaodi Zhang ^4^, Yuanbo Cui ^5^, Jie Pan ^6, *^, Linlin Li ^2, 3, *^, Xin Liu ^1, *^*

^1^ Department of Dental Materials, Shanghai Biomaterials Research & Testing Center, Shanghai Ninth People’s Hospital, Shanghai Jiao Tong University School of Medicine; College of Stomatology, Shanghai Jiao Tong University; National Center for Stomatology; National Clinical Research Center for Oral Diseases; Shanghai Key Laboratory of Stomatology, Shanghai, China

^2^ Beijing Institute of Nanoenergy and Nanosystems, Chinese Academy for Sciences, Beijing, China

^3^ School of Nanoscience and Engineering, University of Chinese Academy of Sciences, Beijing, China

^4^ Institute for Cell Engineering, Department of Neurology, Johns Hopkins University School of Medicine, Baltimore, Maryland, USA

^5^ Leavey School of Business, Santa Clara University, Santa Clara, California, USA

^6^ Department of Orthodontics, Shanghai Stomatological Hospital and School of Stomatology, Fudan University, Shanghai, China

Baiyan Sui, Tingting Ding, and Xingyi Wan contributed equally to this work.

Correspondence:

Xin Liu, Department of Dental Materials, Shanghai Biomaterials Research and Testing Center, Shanghai Ninth People's Hospital, Shanghai Jiao Tong University School of Medicine; College of Stomatology, Shanghai Jiao Tong University; National Center for Stomatology; National Clinical Research Center for Oral Diseases; Shanghai Key Laboratory of Stomatology, Shanghai 200011, China. Email: [liuxin8253@sjtu.edu.cn](mailto://liuxin8253@sjtu.edu.cn)

Linlin Li, Beijing Institute of Nanoenergy and Nanosystems, Chinese Academy for Sciences, Beijing 101400, China. Email: lilinlin@binn.cas.cn

Jie Pan, Department of Orthodontics, Shanghai Stomatological Hospital and School of Stomatology, Fudan University, Shanghai 200001, China. Email: [jiepan@fudan.edu.cn](mailto://jiepan@fudan.edu.cn)


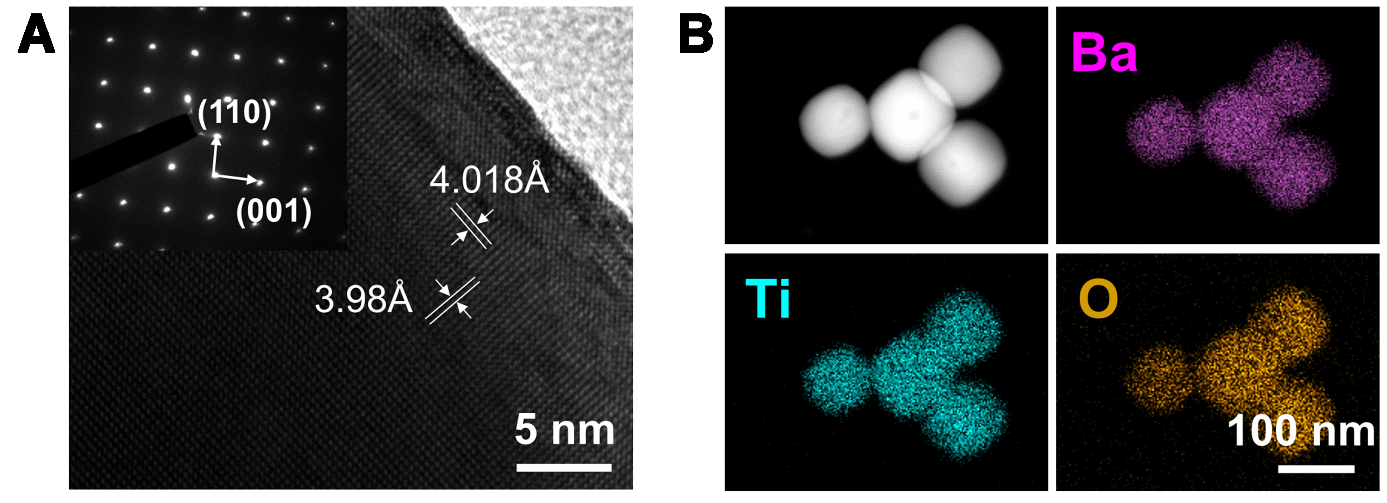


**Figure S1.** (A) HRTEM image of an individual BTO NP and the corresponding SAED pattern (inset), and (B) TEM image and EDS mapping for Ba, Ti, and O.

**
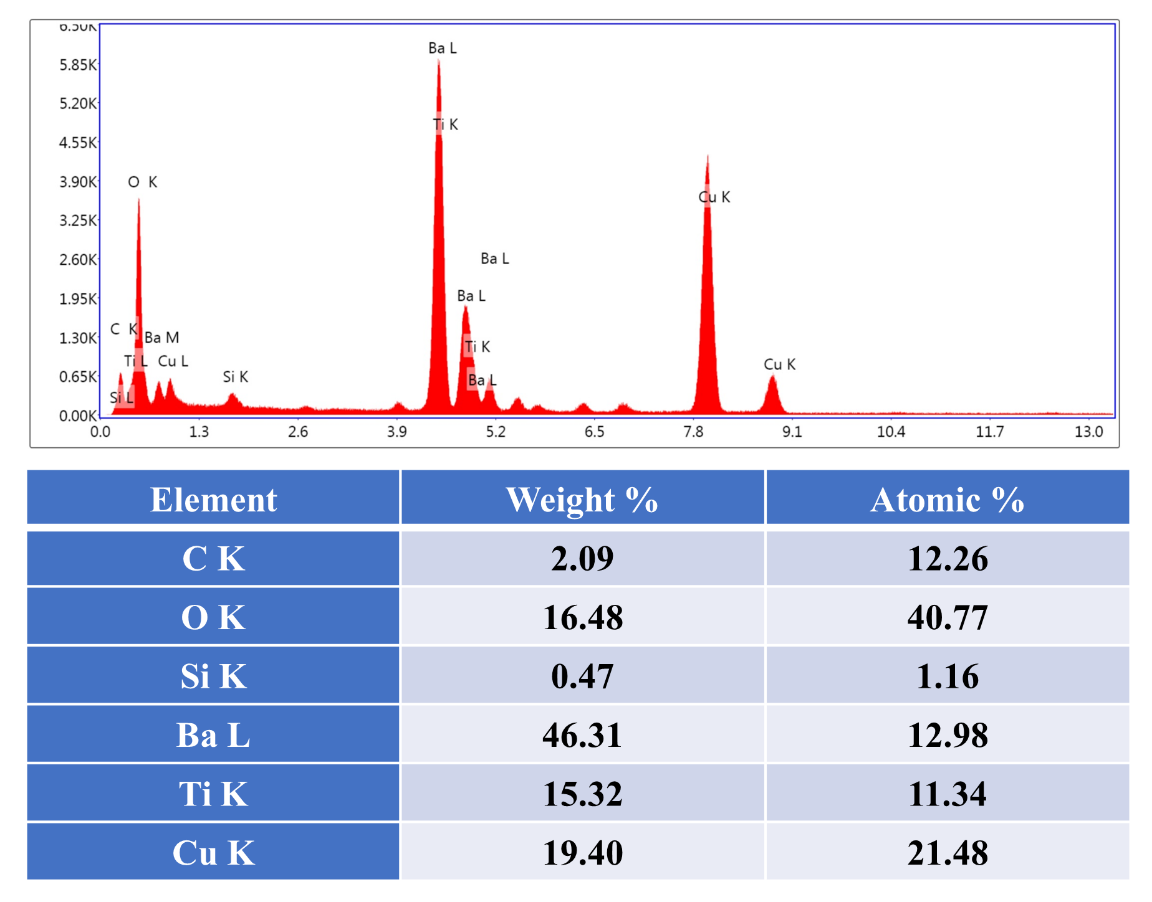
**

**Figure S2.** Energy dispersive X-ray (EDX) spectrum of BTO NPs, and the weight ratio and atom ratio of elements.


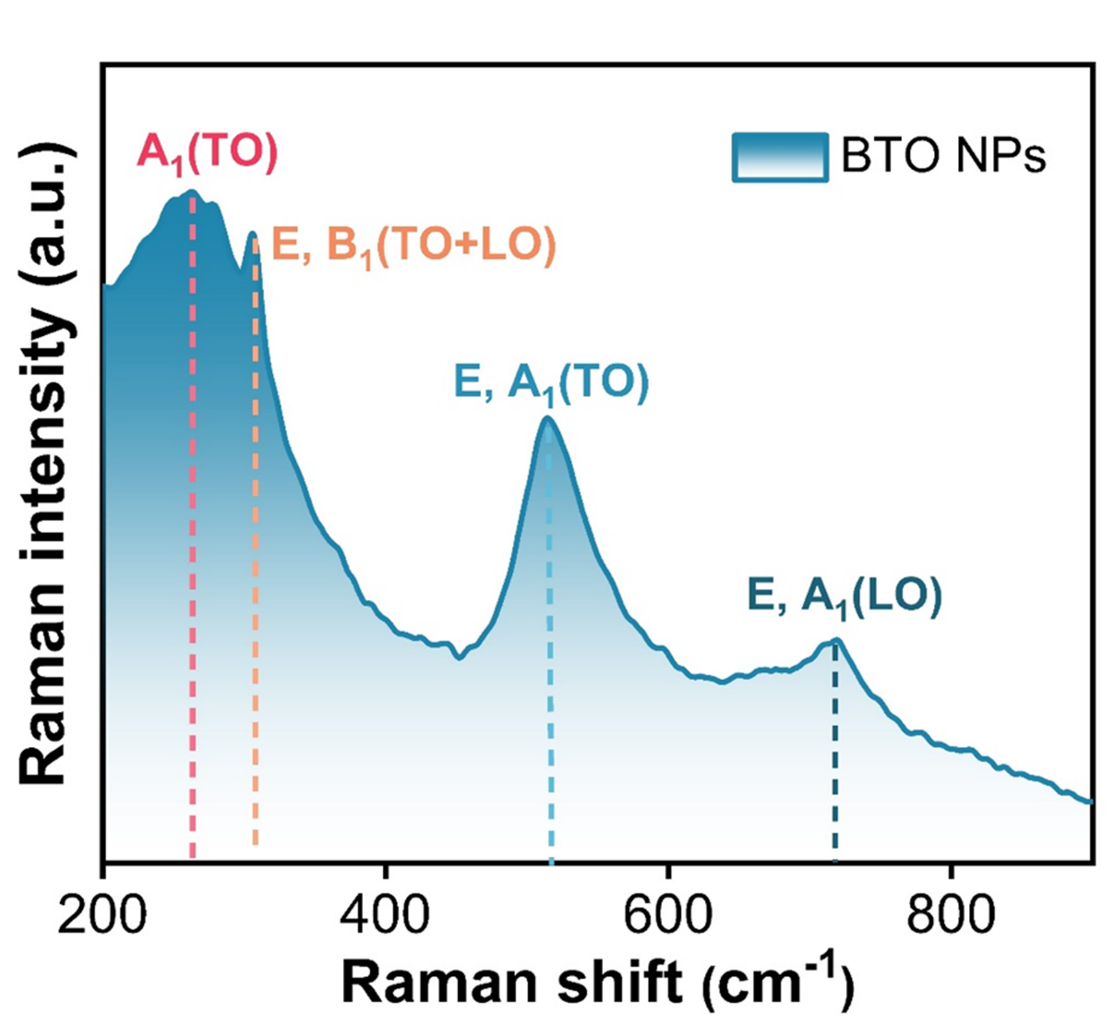


**Figure S3**. Raman spectrum of BTO NPs.


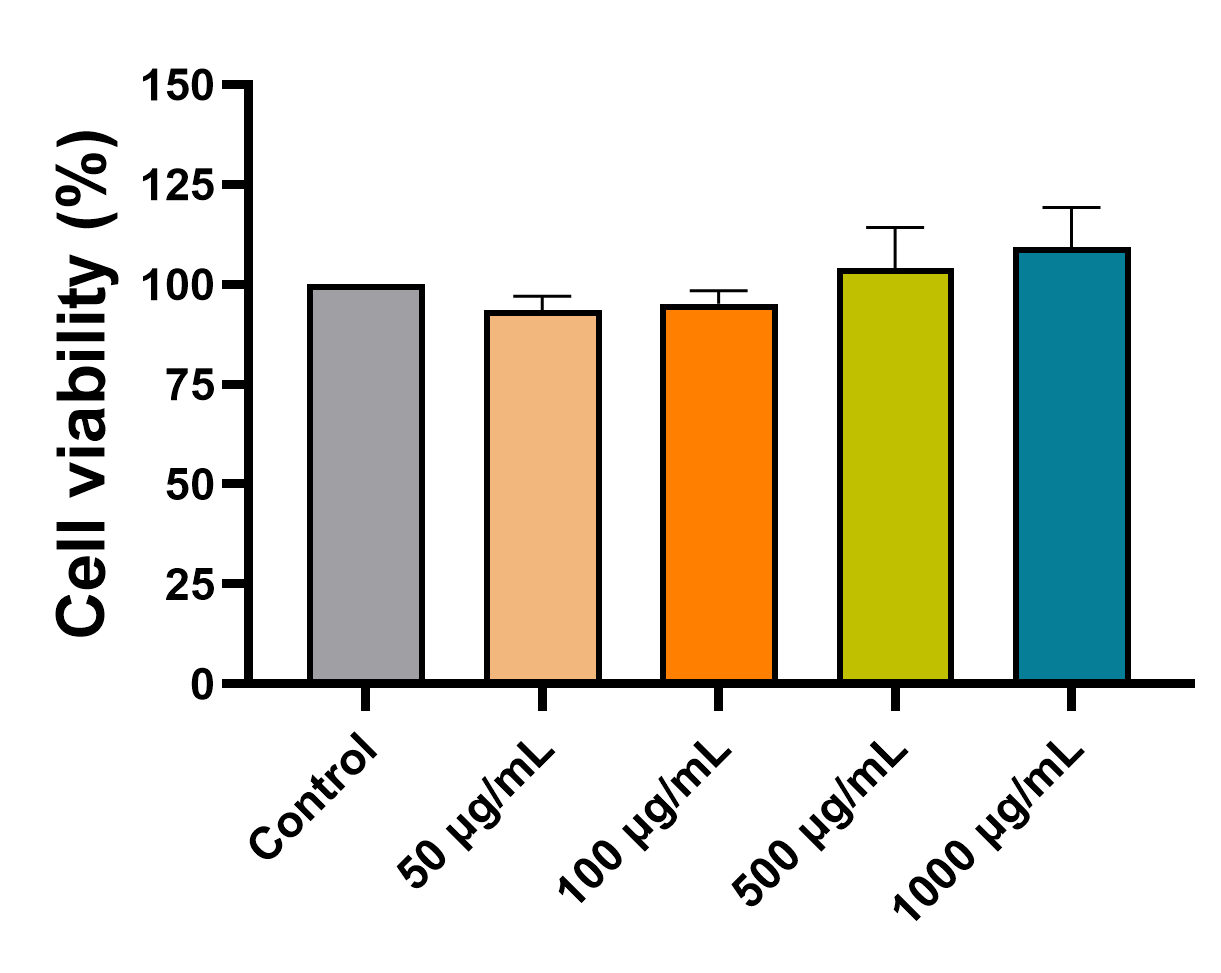


**Figure S4**. Cell viability of BTO NPs on RAW 264.7 cells after 24 h exposure.


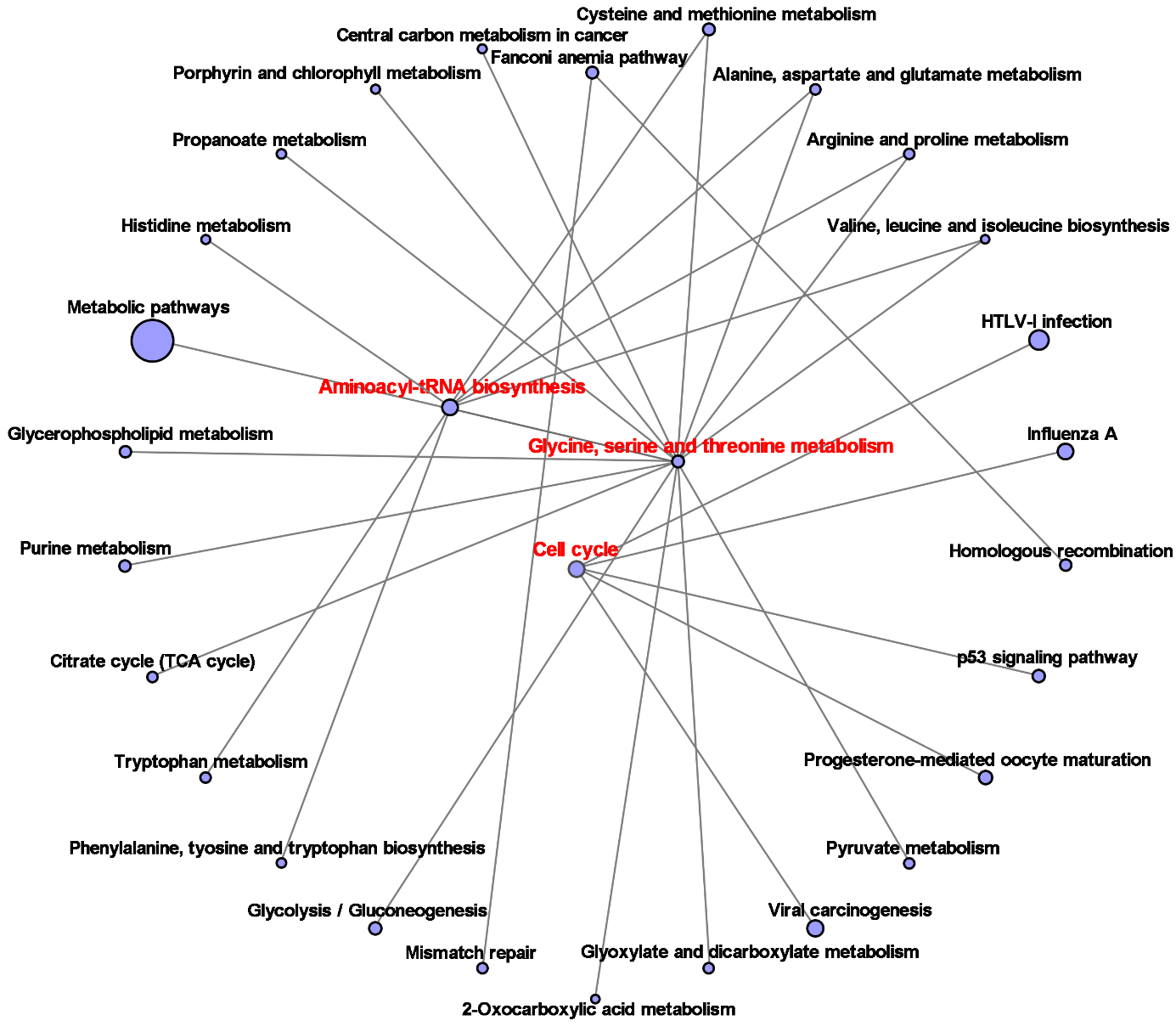


**Figure S5.** Network for the functionally organized KEGG pathway of RAW 264.7 cells between the NC US^-^ group and BTO US^+^ group.


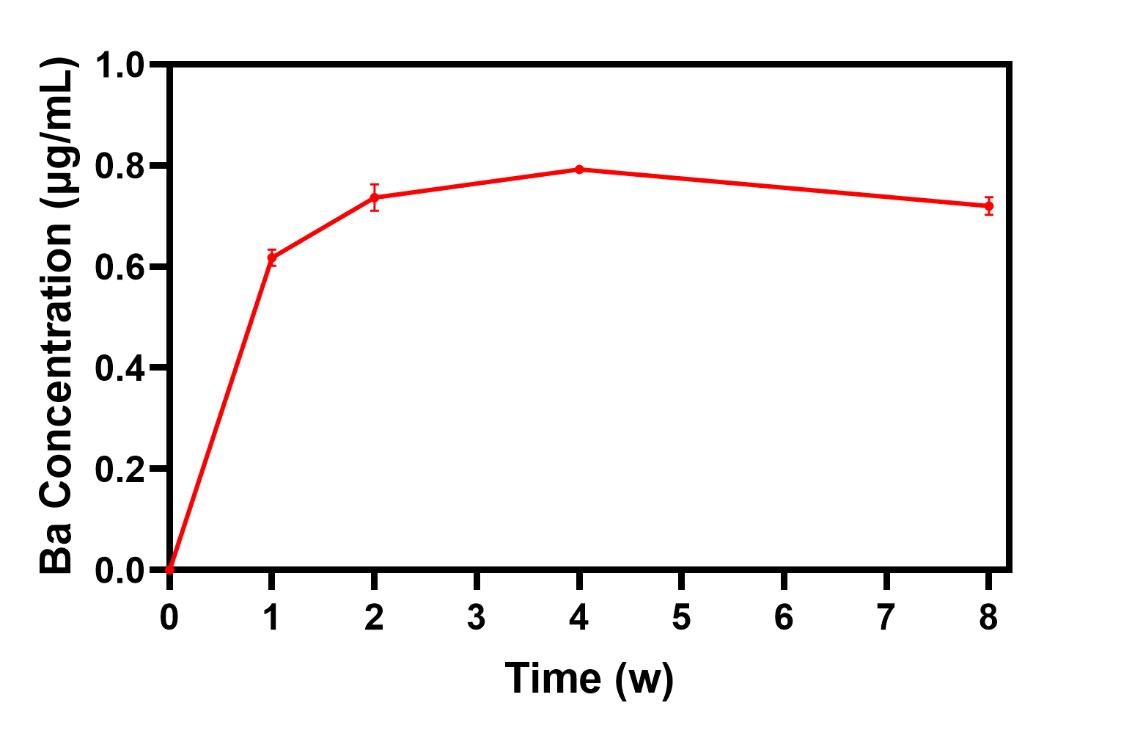


**Figure S6.** Release curve of BTO NPs from GelMA+BTO hydrogel in PBS during 8 weeks.


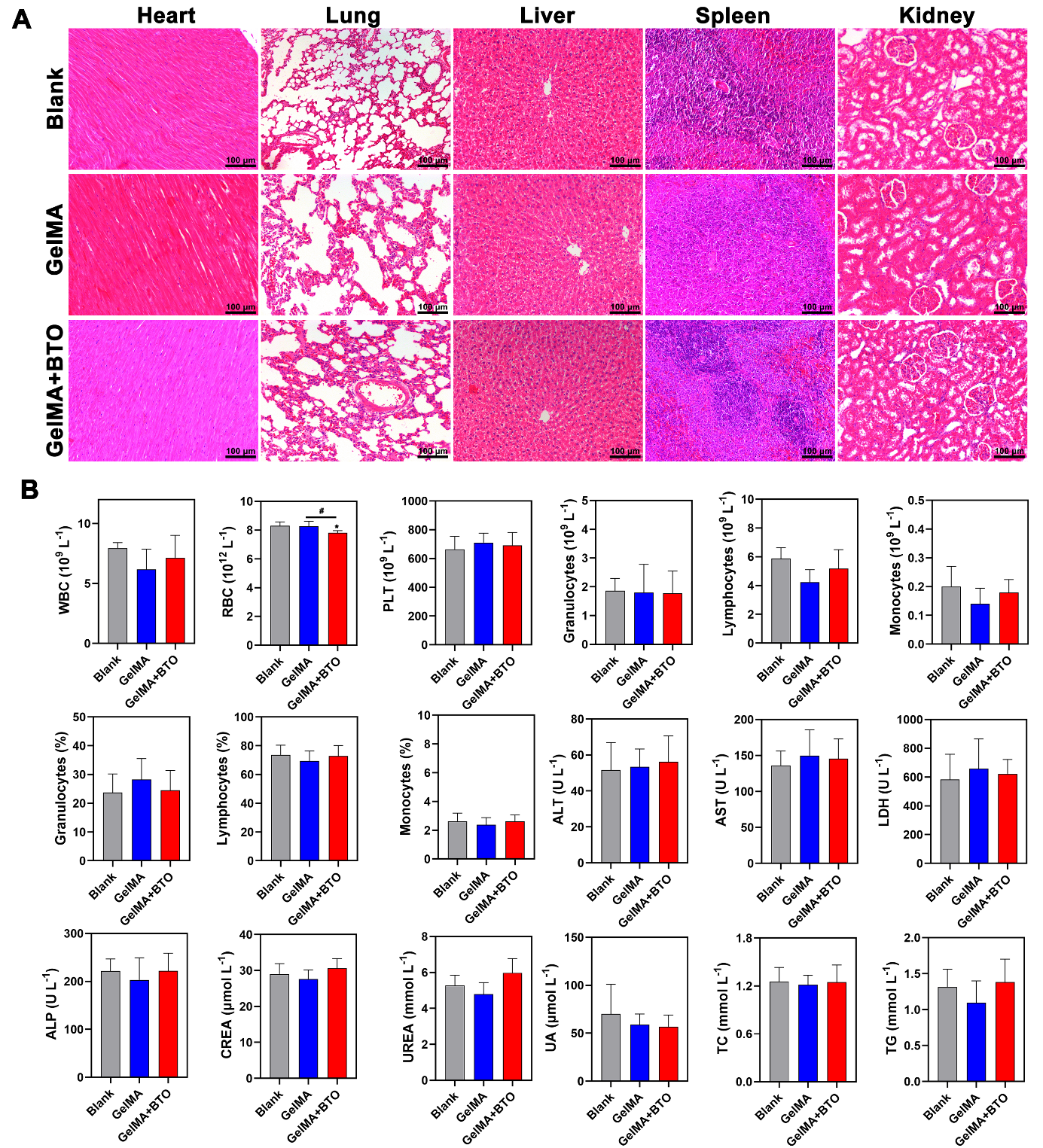


**Figure S7.** *In vivo* bio-safety assessment of BTO-embedded nanocomposite hydrogel after 8w implantation. (A) H&E staining of tissue slices from the major organs for each group. (B) Hematological and biochemical blood indexes for each group, including WBC (10^9^ L^-1^), RBC (10^12^ L^-1^), PLT (10^9^ L^-1^), Granulocytes (10^9^ L^-1^), Lymphocytes (10^9^ L^-1^), Monocytes (10^9^ L^-1^), Granulocytes (%), Lymphocytes (%), Monocytes (%), ALT (U L^-1^), AST (U L^-1^), LDH (U L^-1^), ALP (U L^-1^), CREA (μmol L^-1^), UREA (mmol L^-1^), UA (μmol L^-1^), TC (mmol L^-1^) and TG (mmol L^-1^). *: P < 0.05 versus blank group, #: P < 0.05 compared between GelMA and GelMA+BTO group. n=5 for each group.

**Table S1.** The sequences for RAW 264.7 cells PCR primers

| Gene |  | Primer sequence |
| --- | --- | --- |
| Fizz1 | Forward | 5’- TGGAGAATAAGGTCAAGGAAC-3’ |
|  | Reverse | 5’- GTCAACGAGTAAGCACAGG-3’ |
| Arginase 1 | Forward | 5’- AGACAGCAGAGGAGGTGAAGAG-3’ |
|  | Reverse | 5’- CGAAGCAAGCCAAGGTTAAAGC-3’ |
| iNOS | Forward | 5’- GGCAGCCTGTGAGACCTTTG-3’ |
|  | Reverse | 5’- GCATTGGAAGTGAAGCGTTTC-3’ |
| YM1 | Forward | 5’- CATTCAGTCAGTTATCAGATTCC-3’ |
|  | Reverse | 5’- AGTGAGTAGCAGCCTTGG-3’ |
| GAPDH | Forward | 5’- ACCCAGAAGACTGTGGATGG-3’ |
|  | Reverse | 5’- CACATTGGGGGTAGGAACAC-3’ |

**Table S2.** The sequences for BMSCs PCR primers

| Gene |  | Primer sequence |
| --- | --- | --- |
| Col-1 | Forward | 5’- GCTCCTCTTAGGGGCCACT-3’ |
|  | Reverse | 5’- CCACGTCTCACCATTGGGG-3’ |
| ALP | Forward | 5’- CCAACTCTTTTGTGCCAGAGA-3’ |
|  | Reverse | 5’- GGCTACATTGGTGTTGAGCTTTT-3’ |
| GAPDH | Forward | 5’- AGGTCGGTGTGAACGGATTTG-3’ |
|  | Reverse | 5’- TGTAGACCATGTAGTTGAGGTCA-3’ |
